# A Comprehensive Review of Pea (*Pisum sativum* L.): Chemical Composition, Processing, Health Benefits, and Food Applications

**DOI:** 10.3390/foods12132527

**Published:** 2023-06-29

**Authors:** Ding-Tao Wu, Wen-Xing Li, Jia-Jia Wan, Yi-Chen Hu, Ren-You Gan, Liang Zou

**Affiliations:** 1 Key Laboratory of Coarse Cereal Processing, Ministry of Agriculture and Rural Affairs, Sichuan Engineering & Technology Research Center of Coarse Cereal Industralization, School of Food and Biological Engineering, Chengdu University, Chengdu 610106, China; wudingtao@cdu.edu.cn (D.-T.W.);; 2 Institute for Advanced Study, Chengdu University, Chengdu 610106, China; 3 Singapore Institute of Food and Biotechnology Innovation (SIFBI), Agency for Science, Technology and Research (A*STAR), Singapore 138669, Singapore

**Keywords:** functional properties, bioactive compounds, dietary fiber, polyphenol, modifications, functional grain

## Abstract

*Pisum sativum* L., commonly referred to as dry, green, or field pea, is one of the most common legumes that is popular and economically important. Due to its richness in a variety of nutritional and bioactive ingredients, the consumption of pea has been suggested to be associated with a wide range of health benefits, and there has been increasing focus on its potential as a functional food. However, there have been limited literature reviews concerning the bioactive compounds, health-promoting effects, and potential applications of pea up to now. This review, therefore, summarizes the literature from the last ten years regarding the chemical composition, physicochemical properties, processing, health benefits, and potential applications of pea. Whole peas are rich in macronutrients, including proteins, starches, dietary fiber, and non-starch polysaccharides. In addition, polyphenols, especially flavonoids and phenolic acids, are important bioactive ingredients that are mainly distributed in the pea coats. Anti-nutritional factors, such as phytic acid, lectin, and trypsin inhibitors, may hinder nutrient absorption. Whole pea seeds can be processed by different techniques such as drying, milling, soaking, and cooking to improve their functional properties. In addition, physicochemical and functional properties of pea starches and pea proteins can be improved by chemical, physical, enzymatic, and combined modification methods. Owing to the multiple bioactive ingredients in peas, the pea and its products exhibit various health benefits, such as antioxidant, anti-inflammatory, antimicrobial, anti-renal fibrosis, and regulation of metabolic syndrome effects. Peas have been processed into various products such as pea beverages, germinated pea products, pea flour-incorporated products, pea-based meat alternatives, and encapsulation and packing materials. Furthermore, recommendations are also provided on how to better utilize peas to promote their development as a sustainable and functional grain. Pea and its components can be further developed into more valuable and nutritious products.

## 1. Introduction

**Figure 1:**
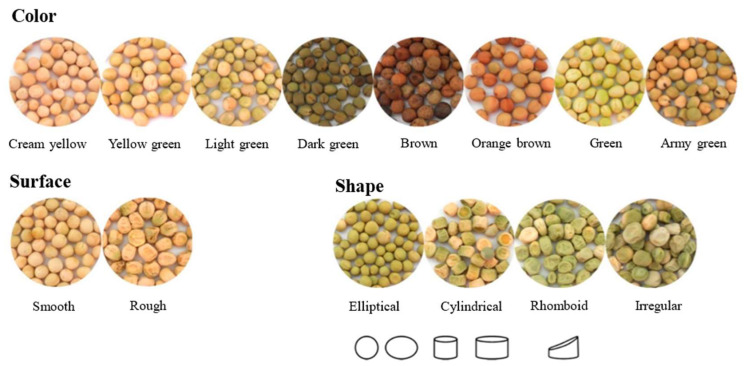
Pea seeds of different surfaces, colors, and shapes [7] (reprinted with permission from the publisher).

*Pisum sativum* L., known as green pea, dry pea, or field pea, is an important legume crop that provides a good source of protein, vitamins, minerals, and bioactive compounds that are beneficial to human health [1,2,3]. Peas are cultivated in almost all countries around the world and regarded as an essential part of the human diet [4]. Canada is the biggest producer of peas around the world, followed by China, Russia, and India [5]. Usually, peas have two phenotypes, namely, the smooth pea and the wrinkled pea, and their seed coats are cream yellow, chartreuse, light green, green, army green, dark green, brown, or orange-brown (Figure 1) [6,7]. The difference observed in the colors of the pea coat is associated with the biosynthesis of flavonoids, which can be affected by different cultivars and grow environment. The content of flavonoids in dark seed coat samples is generally higher than that in light color samples [8]. Peas have a large content of lysine but lack of amino acids with thiol, therefore, they are often consumed together with grains for a full set of essential amino acids [9]. Furthermore, peas can also be consumed as sprouts and microgreens after germination [10,11]. Since 2020, the COVID-19 pandemic has significantly affected the food supply chain, resulting in food self-sufficiency receiving increasing attention [12]. In fact, the sprouts and microgreens derived from pea by germination in-house may be an alternative choice to temporarily solve the shortage of domestic vegetables.

The biological activity and health benefits of pea are usually associated with its nutrients and bioactive ingredients [13]. The glycemic index (GI) of pea is normally lower than 60, thus, it is regarded as a medium- or low-GI food [14]. A recent epidemiological study has shown that a high-GI diet is always related to the increase in risk of cardiovascular diseases [15]. Therefore, the whole seed of pea and its products show good potential to partially replace other high-GI foods. In addition, pea is gluten-free [16], meaning it can be consumed by people who have celiac disease. Pea proteins and peptides possess multiple biological properties [17], such as regulation of metabolic syndrome. Peas are also rich in dietary fiber [18], which can provide various health benefits via regulating gut microbial composition [19]. Peas are a good resource of minerals (e.g., calcium, iron, and zinc) and vitamins (e.g., carotenoids and folic acid) [20]. In addition, this legume also has a large content of polyphenolics, especially flavonoids, which exhibit various biological activities [3,21,22]. Therefore, peas are rich in nutrients and bioactive compounds, and exhibit good potential to be developed into health products or functional foods.

Dietary proteins can be derived from both animals and plants. Although the demand for animal proteins is always high, it is generally considered that animal proteins are less environmentally sustainable [23]. To promote sustainable eating habits, it is encouraged to reduce the reliance on animal-derived proteins. In addition, the intake of proteins from plants may be more beneficial than proteins from animals. For instance, the consumption of proteins from plants is associated with a reduced mortality from all causes and cardiovascular diseases [24]. On the other hand, increasing meat consumption may be associated with obesity, heart disease, metabolic syndrome, and gastrointestinal cancers [25]. Collectively, it can be speculated that the requirement for whole legume-derived products will keep increasing in the future, providing a huge chance for peas to be explored as value-added products. Therefore, a comprehensive understanding of pea can be important for its further application in the food industry. This review summarizes the literature from the last ten years and provides a comprehensive summary and discussion on chemical ingredients and the health benefits of pea and its products. The processing and food application of pea and its components are also highlighted. Finally, the research directions on how to make better use of peas in the future are proposed.

## 2. Chemical Composition of Pea

### 2.1. Proximate Composition

Carbohydrates are regarded as the one of the main chemical components of pea, comprising 59.32–69.59% of the dry weight of pea seeds [26]. The content of starch in pea seeds varies from 39.44% to 46.23% [5], which can be higher than that in faba beans (38.4–41.8%) [27,28]. This pulse is rich in dietary fiber, ranging from 23.23% to 30.72% of pea seeds, with 3.91–8.01% of soluble fiber and 19.32–23.1% of insoluble fiber [18]. This pulse is also rich in proteins, with about 20–25% of the dry weight of pea seeds [29], similar to adzuki beans (23.51%) and kidney beans (23.44–24.90%) [30]. The content of lipids in pea seeds is about 3.06–7.3%, similar to that of cowpea (4.22–7.17%) [18]. The content of ash in pea is about 3.07% [26]. In addition, the cultivar, environment, and planting year significantly influence the nutrients in pea seeds [31]. Nevertheless, to improve the precise use and application of pea in the food industry, more studies are required for the systematic comparison of the chemical composition in its different cultivars.

### 2.2. Starch

Amylose and amylopectin are regarded as the main types of starches, and their ratio remarkably influences the physicochemical properties of starches [32]. The pea starches contain a high content of amylose, in the range of 17.2–42.6%, and the wrinkled pea contains more amylose than the round pea [5,33]. Indeed, starch isolated from wrinkled peas was reported to have longer branch chains of amylopectin than the starch from smooth peas [34]. The pea starches with higher amylose contents may be endowed with increased resistance to digestion [35]. Based on the digestive index, the estimated glycemic index (eGI) of isolated pea starches ranged from 69.8 to 70.7 [36]. Resistant starch (RS) is regarded as one of the most important dietary fiber, which has beneficial effects on human intestinal health [37]. A previous study indicated that the pea seeds contained 1.84–6.95% of RS, significantly higher than that in soybean seeds (0–0.19%) [18]. In addition, different modification methods have been carried out for the preparation of less-digestible starches, which are reviewed and discussed in Section 3.2.

The reported physicochemical characteristics of pea starches have varied according to different studies. The pea starch showed a typical C-type pattern, with the relative crystallinity ranging from 36% to 55%, and the ratio of B-type crystallites ranging from 3.8% to 30.4% [38]. The shapes of pea starch granules are commonly oval or round, as determined by scanning electron microscopy (SEM). However, also some irregularly shaped granules have been reported in pea starches, with granule sizes in the range of 21.5–23.9 μm [39]. A comparative study indicated that the size of white pea starch granules (4.03–21.33 μm) was smaller than pigeon pea starch granules (11.01–56.08 μm), kudzu starch granules (6.8–35.38 μm), and broad bean starch granules (2.07–57.61 μm), suggesting that white pea starches might be more susceptible to acid and enzymatic hydrolysis than the other four legume starches [32]. These structural parameters of pea starches obviously affect their physicochemical properties. The swelling power of starch indicates the water adsorption capacity, and the water solubility reflects the dissolution degree of starch during gelatinization [40]. The solubility and swelling power of white pea starches increased with rising temperature [32]. The peak gelatinization temperature (*Tp*) of pea starches was found in the range of 64.2–70.1 °C. The enthalpy of gelatinization (Δ*H*) of pea starches ranged from 4.67 to 9.2 J/g [41], similar to that of kidney bean starches [42] and black gram starches [43], while lower than that of starches from chickpea [44], lentil [45], green gram [46], faba bean [47], and mung bean [45]. The pasting temperature of starch can reflect the minimum temperature needed for its cooking, and can also indicate the peak viscosity of the paste. Pea starches displayed a similar pasting temperature (66.2–70.1 °C) to that of adzuki bean starches (66.77–69.92 °C) and chickpea starches (66.5–71.1 °C) [39,44,48]. The peak viscosity of starches in smooth peas was measured to be 2909 cP in a previous study, which was higher than that of kidney bean starches (2245 cP), red adzuki bean starches (2316 cP), and cowpea starches (1689 cP) [49]. The relatively high viscosity of pea starches can contribute to their food textural characteristics. Nevertheless, more studies should be carried out for verifying these results, since the physicochemical characteristics of starches can be influenced by different factors.

### 2.3. Dietary Fiber

Dietary fiber, as the main non-digestible carbohydrates, can provide various health benefits via regulating gut microbial composition [19]. Dietary fiber is commonly divided into soluble dietary fiber (SDF) and insoluble dietary fiber (IDF) based on its water solubility. Pea seeds are rich in dietary fiber, which range from 23.23% to 30.72%, with 3.91–8.01% of SDFs and 19.32–23.1% of IDFs [18]. A comparative study indicated that the contents of SDFs in pea seeds were similar to those in broad beans (4.89–5.05%), white kidney beans (4.57–5.14%), cowpeas (4.23–5.82%), red beans (5.04–5.59%), and black soybeans (6.59–8.11%) [18]. Indeed, the contents of IDFs in pea seeds were also comparable to those in soybeans (18.28–21.99%), mung beans (17.92–20.17%), red beans (20.41–24.73%), and cowpeas (17.89–22.33%), while lower than those in white kidney beans (24.73–26.75%), red kidney beans (26.52–26.96%), lentil seeds (23.57–24.93%), and broad beans (26.85–28.69%) [18]. In addition, the content of SDFs in pea seeds could be improved by ultrafine grinding technology, which increased the content from 1.26% to 4.97% [50]. Generally, foods containing a high content of dietary fiber can lower serum cholesterol and glycemic indexes in vivo [19,51]. Therefore, pea may be a dietary source for the prevention of diabetes and hypercholesterolemia.

SDFs in pea seeds are composed of galacturonic acid, arabinose, galactose, glucose, mannose, rhamnose, xylose, and fucose [52,53], with galacturonic acid as the predominant sugar [52], indicating that pea SDFs contain a large number of pectic polysaccharides. The sugar compositions of IDFs in pea seeds include glucose, arabinose, galacturonic acid, xylose, galactose, mannose, and rhamnose, with glucose, xylose, and arabinose as the major sugars [52], suggesting that pea IDFs should contain cellulose, xylans, and arabinans. The molecular weights of SDFs in pea seeds were reported to be in the range of 25–478 kDa [52,53], and their intrinsic viscosities and apparent viscosities ranged from 0.84 to 0.85 dL/g and 1.73 to 1.87 mPa·s, respectively [52]. The microstructures of polysaccharide fractions isolated from pea seeds showed a smooth surface [54]. In addition, pea dietary fiber and polysaccharides exhibited remarkable antioxidant activity in vitro [50,53,55] and hypoglycemic effects in vivo [46,49,51]. Nevertheless, the knowledge regarding the detailed chemical structures of SDFs and dietary polysaccharides is still not clear, and requires further clarification.

### 2.4. Protein

Pea protein is commonly classified into four categories, namely, globulin, albumin, prolamin, and glutenin, of which globulin is the main storage protein, accounting for about 55–65% of the total protein in field peas [56]. Pea protein primarily consists of 7S/11S globulin and 2S albumin, and has a large content of lysine, which can make up for the lack of lysine in cereal-based diets [17,56]. Pea protein and its hydrolysates possess several health-promoting effects, such as antioxidant, anti-diabetic, and anti-hypertensive effects, as well as regulation of intestinal microbial composition [17]. In addition, pea proteins are widely used in the food system, such as in encapsulation for bioactive compounds, degradable films, and as an alternative to animal proteins [17]. Generally, changes in the ratio of globulin to albumin and soy protein to vecillin may affect the presence and intensity of potential allergens [57]. Several allergenic proteins have been found in peas, such as pea 7S globulin Pis s 1, which has been identified as a main immunodominant allergen for pea-allergic children [58]. Pis s 2, a contaminant in the globulin component vecillin, was also found as a pea allergen. Furthermore, the albumin component also contained two less well-characterized allergic proteins, namely, PA1 and PA2 [59]. At present, studies on the allergenic properties of peas are still incomplete, and the effects of food processing on pea allergies are not well understood.

The amino acid composition of pea proteins is well equilibrated, being rich in different essential amino acids. Two amino acids, methionine and cysteine, are considered as the main limiting amino acids (LACs) of pea seeds [30], similar to other beans. However, when using the methodology based on true ileal amino acid digestibility, the main LACs for infants in cooked peas are aromatic amino acids, while for older children, adolescents, and adults, the main LAC in cooked peas is lysine [60]. Consequently, other sources of protein can be utilized to make up for the lacking of indispensable amino acids in peas to achieve a nutritionally balanced diet. Furthermore, a recent study reported that some physicochemical characteristics of pea proteins are very close to soybean proteins, making them a promising soybean protein substitute [61]. Both pea and soybean proteins exhibit a typical “U shape” in the curve of pH-dependent solubility, but it is not entirely perfect in an acidic environment that is lower than pH 5. The isoelectric point of the pea protein (pH 4–5) is very close to that of the soybean protein (pH 4–6) [61]. In addition, high-pressure processing and heat treatment obviously reduced the solubility of pea proteins [62]. The foaming stability of pea proteins was about 89.74%, similar to that of soybean proteins (82.44%) and obviously higher than that of rice proteins (50%) and wheat proteins (68.03%) [61]. The water adsorption capacity of pea proteins was about 3.389 g/g, which was higher than that of rice proteins (1.46 g/g) and wheat proteins (1.376 g/g). The least gelation concentration of pea proteins was 14%, similar to that of soybean proteins (12%). The emulsifying activity index and emulsion stability index of pea proteins were similar to those of soybean proteins, suggesting that pea proteins can be a potential alternative to soybean proteins in the production of meat and sausage products [61]. Nevertheless, due to the limited sample scale and limited genotypes, these comparative data may be not very accurate or conclusive and need further systematical investigation.

### 2.5. Lipids

The content of lipids in pea seeds is relatively low, making the pea a low-fat food. Pea lipids predominantly consist of polyunsaturated fatty acids, ranging from 42.01% to 60.68% of total fatty acids, with a relatively low content of unsaturated fatty acids (17.46–24.95%) [63]. The fatty acids of peas mainly include palmitic acid (12.39–19.24%), linoleic acid (34.56–47.74%), and linolenic acid (7.37–12.55%) [63]. The bioavailability of these unsaturated fatty acids during gastrointestinal digestion is not well known. Furthermore, more studies are required to confirm these results.

### 2.6. Minerals and Vitamins

The pea is a potential resource of several minerals (e.g., nitrogen, potassium, and phosphorus). It was observed that the mineral element content (e.g., nitrogen, potassium, phosphorus, manganese, copper, and zinc) varied among different genotypes of pea seeds [64]. Nitrogen (28.49–54.78 g/kg), phosphorus (1.648–4.04 g/kg), and potassium (13.13–50.41 g/kg) were found as the major minerals in pea seeds [64]. In addition, copper (3.51–21.79 mg/kg), iron (29.32–80.69 mg/kg), zinc (28.15–55.80 mg/kg), and manganese (7.96–22.83 mg/kg) also varied among different genotypes of pea seeds. A minor amount of selenium was also found in pea seeds (28.6 μg/100 g) [65], but the content was still obviously higher than that in mung beans. The bioavailability of these minerals in peas remains unclear, which should be further investigated. Furthermore, several vitamins are also found in peas, such as α-tocopherol and γ-tocopherol. The contents of total tocopherols in pea seeds varied among different cultivars, ranging from 48.44 to 57.00 μg/g, higher than in lentils (29.65–46.18 μg/g) and kidney beans (22.53–35.82 μg/g), while lower than in chickpeas (150.29–170.51 g/g) [66]. In addition, γ-tocopherol can be the major tocopherols in pea seeds, ranging from 46.14 to 54.17 μg/g. Nevertheless, more studies are still required to confirm these results. Furthermore, whether food processing can affect the bioavailability of minerals and vitamins is still unclear.

### 2.7. Polyphenols

#### 2.7.1. Total Phenolic Content

Phenolics are regarded as one of the most important bioactive components in peas. Both free and bound polyphenols are found in peas. A previous study revealed that the content of free phenolics (90.4–112 mg GAE/100 g DW) in three genotypes of peas were higher than that of bound phenolics (58.5–83.9 mg GAE/100 g DW) [67]. Furthermore, total polyphenols of 22 different pea genotypes, including different maturities, different flower colors, different seed coat colors, and different seed shapes, were systematically investigated. It was revealed that the total phenolic content (TPC) in peas varied in 22 genotypes, ranging from 12.6 to 128.6 mg GAE/100 g FW, which was significantly correlated with their color and shape of seed coats [8]. The genotypes exhibiting greenish-orange colored seed coats possessed the highest TPC (128.6 mg GAE/100 g FW) among all genotypes. On the other hand, the genotypes exhibiting dimpled and round seed coats also possessed obviously higher TPC (≥24.0 mg GAE/100 g FW) than that of those with wrinkled seed coats [8]. In addition, dark-colored seeds had higher TPC than light-colored seeds [68]. Furthermore, the colored hulls of pea seeds also possessed phenolic compounds [69]. During in vitro digestion, the TPC released from the red hulls of peas was about 31.54 ± 0.69 mg GAE/g DW, higher than in the yellow hulls (14.88 ± 0.27 mg GAE/g DW) [69]. Moreover, the TPC in pea sprouts obviously increased from 584.32 to 910.69 mg GAE/100 g DW after germination for seven days [70], suggesting that germination can improve the content of polyphenols in peas.

#### 2.7.2. Flavonoids

Different flavonoids, including flavonols, flavones, isoflavones, flavanones, flavanols/flavan-3-ols, and anthocyanins (Table 1), have been determined in different parts of peas using multiple techniques, such as liquid chromatography (LC), LC coupled with mass spectrometry (LC-MS), LC coupled with electrospray ionization-tandem mass spectrometry (LC-ESI-MS/MS), and ultra-HPLC coupled with quadrupole orbitrap high-resolution mass spectrometers (UHPLC-Q-HRMS) [21]. The total flavonoid content (TFC) in peas ranged from 4.61 to 45.84 mg CE/100 g FW, with a nearly 10-fold variation [8]. In addition, the content of soluble flavonoids (52.2–60.3 mg CE/100 g DW) is higher than bound flavonoids (8.42–20.3 mg CE/100 g DW) in pea seeds [67]. Interestingly, the TFC in pea seeds was significantly correlated with the color and the shape of the seed coats [8]. A higher TFC was found (≥9 mg CE/100 g FW) in the genotypes exhibiting dimpled and round seed coats, and the genotypes exhibiting greenish-orange colored seed coats possessed the highest TFC among all tested genotypes [8]. It was found that pea seeds with dark colors contained more TFC than light-colored pea seeds [68]. Furthermore, the germination treatment could also increase the TFC in pea sprouts, increasing from 4.53 to 6.02 mg CE/100 g DW [70].

Glycosylated flavonols are found as the major phenolic compounds in peas [21]. Many new flavonoid compounds have also been identified in different parts of peas in recent years (Table 1). For instance, several new flavonols, such as quercetin 3-galattoside, quercetin diglucoside, quercetin triglucoside, quercetin caffeoyl triglucoside, quercetin coumaroyl triglucoside, quercetin sinapoyl triglucoside, quercetin feruloyl triglucoside, dihydrokaempferol, kaempferol-3-O-rhamnoside, kaempferol-7-O-glucoside, kaempferol-7-O-rutinoside, kaempferol triglucoside, kaempferol hexoside, kaempferol dihexoside, kaempferol coumaroyl, myricetin 3-O-rhamnoside, dihydromyricetin, and isorhamnetin 3-rutinoside, have been discovered in pea seeds, seed coats, pods, sprouts, and leaves by several different techniques, such as LC-MS, UHPLC-MS, UHPLC-Q-HRMS, and UHPLC-linear ion-trap quadrupole (LTQ)-OrbiTrap-MS analysis (UPLC-LTQ-MS) [71,72,73]. Several new flavones have also been determined in pea seeds and seed coats, such as apigenin-7-O-glucoside, luteolin 8′-O-glucoside, luteolin 3′,7-di-O-glucoside, and luteolin-8′-C-glucoside [74,75,76]. Three new flavanones, including naringin, melitidin, and eriodictyol, were found in pea seeds and seed coats [74,75,77]. It was also found that flavan-3-ols (e.g., epigallocatechin and gallocatechin) and flavonols (e.g., myricetin-3-O-rhamnoside and quercetin-3-O-rhamnoside) existed in obviously higher concentrations (1300–6100 times) in seed coats of the purple flower pea line than in the white flower pea line [76]. In addition, some isoflavones have also been isolated from pea pods, seeds, and seed coats, such as genistein, myricetin, prunetin, isoformononetin, and daidzein [21,71,76]. Furthermore, three new anthocyanins, including pelargonadin 3-glucoside, cyanidin 3,5-di-O-glucoside, and malvidine-3-O-glucoside, have also been identified in pea seeds and pods [76,78]. Nevertheless, detailed structural information regarding several glycosylated flavonols requires further elucidation.

**Table 1:** Bioactive compounds isolated from different pea raw materials and derived products, together with their identification methodology.

Family	Compounds	Plant Part	Methods	References
Flavonols	Isorhamnetin 3-rutinoside, isorhamnetin glycoside, quercetin, quercetin 3-galattoside, rutin, quercetin triglucoside, quercetin diglucoside, kaempferol triglucoside, quercetin caffeoyl triglucoside, quercetin coumaroyl triglucoside, quercetin sinapoyl triglucoside, quercetin feruloyl triglucoside, isorhamnetin glycoside, kaempferol glucoside, kaempferol coumaroyl, kaempferol, dihydromyricetin, kaempferol 3-O-rutinoside-4′-glucoside, dihydroquercetin, myricetin 3-O-rhamnoside, kaempferol 3-O-glucoside, kaempferol hexoside, kaempferol-7-O-glucoside, kaempferol-7-O-rutinoside, kaempferol-3-O-rhamnoside, kaempferol dihexoside, isorhamnetin, dihydrokaempferol, kaempferol 3-O-glucopyranoside, fisetin, kaempferol 3-O-neohesperidoside, kaempferol 3-O-sophorotrioside, kaempferol 3-O-(6″″-O-trans-p-coumaroyl)-sophorotrioside, galangin, morin, quercetin 3-O-β-D-glucopyranoside, quercetin 3-O-sophorotrioside, quercetin 3-O-(6″″-O-trans-p-coumaroyl)-sophorotrioside, quercetin 3-O-(6″″-O-trans-caffeoyl)-sophorotrioside, quercetin 3-O-(6″″-O-trans-feruloyl)-sophorotrioside, quercetin 3-O-(6″″-O-trans-sinapoyl)-sophorotrioside, quercetin 3-O-(6″″-O-(4-hydroxy)-trans-cinnamoyl)-sophorotrioside, Pisumflflavonoside II [quercetin 3-O-(6″″-O-trans-p-coumaroyl)-sophorotrio-side 7-O-β-D-glucopyranoside], Pisumflflavonoside II [quercetin 3-O-(6″″-O-trans-p-coumaroyl)-sophorotrio- side 7-O-β-D-glucopyranoside]	Seed, seed coat, pod, sprout, leaf	LC-MS, LC-ESI-MS, LC-ESI-MS/MS, UHPLC-MS, UHPLC-LTQ-MS, UHPLC-Q-HRMS	[21,71,72,73,74,75,77,78,79,80]
Flavones	Phloretin, apigenin, luteolin-7-O-glucoside, eriodictyol glycoside, apigenin-7-O-glucoside, luteolin, luteolin 8′-O-glucoside, vitexin, luteolin 3′,7-di-O-glucoside, apigenin-6.8-di-C-glucoside, luteolin-8′-C-glucoside, tricin	Seed, seed coat, pod	LC-MS, LC-ESI-MS, LC-ESI-MS/MS, UHPLC-MS	[21,71,72,74,75,76,78]
Flavanols	Catechin, (epi) catechin, gallocatechin, (epi) gallocatechin, fisetin, catechin gallate	Seed, seed coat, pod, sprout	LC-MS, LC-ESI-MS/MS, UHPLC-MS, UHPLC-LTQ-MS, UHPLC-Q-HRMS	[21,71,73,75,76,77,78,79]
Flavanones	Eriodictyol, naringenin, naringin, hesperidin, melitidin, pinocembrin, liquiritigenin, hesperetin	Seed, seed coat, pod, sprout	LC-MS, LC-ESI-MS, UHPLC-MS, UHPLC-Q-HRMS, UHPLC-LTQ-MS	[21,72,74,75,76,77,79]
Isoflavones	Genistein, daidzein, cirsiliol, prunetin, afrormosina, formononetin, isoformononetin, pseudobaptigenina, sayanedin,	Seed, seed coat, pod, sprout	LC-ESI-MS, UHPLC-MS, UHPLC-LTQ-MS	[21,71,74,75,79]
Anthocyanins	Cyanidin 3-sambubioside-5-glucoside, cyanidin 3-sophoroside-5-glucoside, delphinidin 3-sambubioside-5-glucoside, delphinidin 3-sophoroside-5-glucoside, delphinidin 3-O-(2-O-β-D-xylopyranosyl-β-D-galactopyranoside)-5-O-β-D-glucopyranoside, delphinidin 3-O-(2-O-β-D-xylopyranosyl-β-D-galactopyranoside)-5-O-(6-O-acetyl)-β-D-glucopyranoside, pelargonadin 3-glucoside, cyanidin 3,5-di-O-glucoside, malvidine-3-O-glucoside,	Seed, seed coat, pod	LC-MS, UHPLC-MS	[21,75,76]
Phenolic acids	gallic acid, vanillin, syringic acid, quinic acid, protocatechuic acid, chlorogenic acid, 4-o-caffeoylquinic acid, p-coumaric acid, trans-ferulic acid, trans-cinnamic acid, p-hydroxybenzoic acid, dicaffeoyl quinic acid, caffeic acid, 3,4-dihydroxybenzoic acid, 4-hydroxybenzoic acid, vanillin acid, ferulic acid, coumaroyl quinic acid, 5-feruloylquinic acid, vanillic acid-4-β-D-glucoside, cinnamic acid, o-coumaric acid, 2,3-dihydroxybenzoic acid, 3,4-dihydroxybenzoic acid, ferulic acid, gentisic acid, m-hydroxybenzoic acid, p-hydroxybenzoic acid, 4-hydroxy-3-methoxybenzoic acid, p-hydroxyphenylacetic acid, rosmarinic acid, salicylic acid, sinapic acid, tannic acid, veratric acid	Seed, seed coat, pod, sprout	LC-MS, LC-ESI-MS/MS, LC-ESI-MS, UHPLC-MS, UHPLC-LTQ-MS	[21,70,73,74,75,76,78,80]

ESI, electrospray ionization; HRMS, high-resolution mass spectrometry; LC, liquid chromatography; LTQ, linear ion-trap quadrupole; MS, mass spectrometry; MS/MS, tandem mass spectrometry; Q, quadrupole; UHPLC, ultrahigh-performance liquid chromatography.

#### 2.7.3. Phenolic Acids

Phenolic acids are the second largest class of polyphenols in peas followed by flavonoids [21]. It was found that the colored pea seed coats contained a higher content (78.53 g/g DW) of phenolic acids than the corresponding white pea seed coats (17.17 g/g DW), among which colored seed coats mainly contained vanillic acid, gentisic acid, and protocatechuic acid, while the white seed coats mainly contained ferulic acid and coumaric acid [81]. Recently, various new phenolic acids, such as vanillin acid, quinic acid, coumaroyl quinic acid, 5-feruloylquinic acid, 4-O-caffeoylquinic acid, trans-ferulic acid, trans-cinnamic acid, p-hydroxybenzoic acid, and 4-hydroxybenzoic acid, have also been identified in different parts of peas [73,74,75,76,78], which are listed in Table 1. In particular, it was found that syringic acid was absent in any of the seed coats, while gallic acid and caffeic acid existed in the seed coats of purple flower lines [76]. However, another study found the presence of syringic acid in the yellow pea shell [73]. It was found that the total amount of phenolic acids in the water-based extract of pea pods was 73.15 mg/100 g, and 5-caffeoylquinic acid was the phenolic acid with the highest level in the extract, with a mean value of 59.87 mg/100 g [71]. In addition, the concentrations of gallic acid, ferulic acid, and syringic acid in pea seeds increased significantly during germination [70]. In general, the types and contents of phenolic acids in peas varied among the plant parts, colors, and extraction methods.

### 2.8. Other Beneficial Components

Other beneficial compounds are also present in peas, such as β-carotene and zeaxanthin. A comparative study found that the contents of total carotenoids varied widely in different pea varieties, ranging from 16.72 to 59.39 mg β-carotene/kg DW [82]. In addition, the content of carotenoids in green cotyledons of peas was found to be 10–27 μg/g DW, which was slightly higher than that in yellow cotyledons (5–17 μg/g DW) [20]. Indeed, the mean concentrations of lutein, β-carotene, zeaxanthin, and violaxanthin in 94 pea accessions were measured to be 11.2 μg/g, 0.5 μg/g, 0.3 μg/g, and 0.3 μg/g, respectively [20]. The existence of other beneficial compounds in peas remains to be investigated in the future.

### 2.9. Anti-Nutritional Factors

Due to the existence of a large content of various anti-nutritional factors in legumes, their nutritional quality and beneficial effects are always challenged. Usually, tannins, phytic acid, cyanogenic glycosides, saponins, oxalates, biogenic amines, lectins, protease inhibitors, and α-amylase inhibitors are considered as legume anti-nutritional factors [83,84]. Regarding peas, phytic acid, lectins, oxalates, and trypsin inhibitors have been found as the main anti-nutritional factors [9,85].

Phytic acid is generally considered an anti-nutritional factor in peas. Its anti-nutritional properties are attributed to the formation of insoluble complexes with minerals (e.g., copper, iron, and zinc), resulting in the reduction in their absorption in the human gastrointestinal tract [9]. For example, it was a significant inhibitor of in vitro iron absorption in mature peas, whereas iron bioavailability was higher in immature peas [86]. Its content in peas (8.55–12.40 mg/g DW) was similar to that in lentils (8.56–15.56 mg/g DW) and chickpea (11.33–14.00 mg/g DW), but lower than that in faba beans (19.65–22.85 mg/g DW), common beans (15.64–18.82 mg/g DW), and soybeans (22.91–35.9 mg/g DW) [9,87]. Some processing methods, such as soaking, roasting, boiling, pressure cooking, and sprouting, are effective in decreasing anti-nutritional factors, and the combined application of soaking, roasting, and pressure cooking can be the most effective choice to reduce the content of phytic acid [88]. In addition, the level of lectins in peas (5.53–5.64 hemagglutinin unit/mg DW) was similar to that in faba beans (5.52–5.55 hemagglutinin unit/mg DW), but significantly lower than that in red kidney beans (88.52 hemagglutinin unit/mg DW), soybeans (692.82 hemagglutinin unit/mg DW), and lentils (10.91–11.07 hemagglutinin unit/mg DW) [9]. Indeed, the cooking method can notably decrease the level of lectins in peas [9]. Furthermore, the content of total oxalates in peas (244.65–293.97 mg/100 g DW) was similar to that in faba beans (241.5–291.42 mg/100 g DW), while lower than that in soybeans (370.49 mg/100 g DW). Both cooking and soaking methods can obviously decrease the level of total oxalates in peas [9]. In addition, the content of tannins in peas was determined to be 161.26 mg/100 g DW, similar to that in chickpeas (165.68 mg/100 g DW), but significantly lower than that in lentils (282.3 mg/100 g DW) and common beans (410.93 mg/100 g DW) [89]. Usually, tannins are complex phenolic compounds, which can reduce the bioavailability of nutrients in gut [90]. However, many studies have revealed that tannins possess numerous health-promoting effects, such as antioxidant, anti-diabetic, anti-inflammatory, anti-cancer, anti-allergic, and antimicrobial effects [90,91]. Although the food applications of tannins are limited, they have been widely utilized in the pharmaceutical industry.

On the other hand, peas also contain trypsin inhibitors [85,89], which can obviously affect the activities of trypsin and chymotrypsin, and further impact protein digestion in living bodies [92]. The trypsin-inhibitory activity of peas was 2.27 TIU/g, similar to that of faba beans (2.84 TIU/g) and lentils (2.71 TIU/g), and significantly lower than that of chickpeas (7.14 TIU/g) and common soybeans (16.22 TIU/g) [89]. In addition, the chymotrypsin-inhibitory activity of peas was 3.61 CIU/mg, which was significantly lower than that of common beans (27.15 CIU/mg). Furthermore, a previous study showed that heat processing, such as cold-pelleting and extruding, could obviously reduce the trypsin-inhibitory activity of peas [85]. In the future, more studies should be performed to clarify the impacts of different food processing techniques on pea anti-nutritional factors.

## 3. Processing of Pea and Its Components

Different processing techniques have been applied to process peas and their components, thereby expanding their applications in the food industry [93]. This section describes various processing techniques, such as drying, milling, soaking, cooking, and other methods to improve the functional properties of peas. Considering the chemical compositions of peas, this section also discusses techniques for altering the physicochemical properties of pea starches and proteins.

### 3.1. Processing of the Whole Pea Seeds

#### 3.1.1. Drying

Usually, the harvested fresh seeds are susceptible to germination or mildewing. Therefore, the drying technique can be a potential way to reduce the post-harvest losses. The drying temperature possesses a remarkable effect on the final quality of peas. Thermal treatment at 55 °C was reported as the most suitable temperature, as it did not exhibit any peeling or loss in flavor of the peas [94]. In addition, the ultrasound-assisted heat pump intermittent drying (UHPID) technique is relatively slow in the drying process compared to other drying methods, but it can notably decrease energy expenditure and promote seed vigor to a certain extent [95]. It was found that the comprehensive drying performance of pea seeds was the best under the conditions of a drying temperature of 36 °C, ultrasound power of 200 W, and intermittency ratio of 0.5 [95]. Furthermore, ultrasound application significantly improved the enzymatic activities of superoxide dismutase, peroxidase, and catalase, and reduced the content of malondialdehyde, which could improve the vigor of seeds [96]. On the other hand, the germination percentage of peas decreased with a heating-based drying process, decreasing from 96.0% to 84.0% as the drying temperature increased from 30 °C to 40 °C [96]. In addition, a high ultrasound power was found to be more helpful to increase the germination percentage and the germination index, and decrease the mean germination time of seeds [96]. Nevertheless, how the drying conditions (e.g., temperature, duration, and equipment) impact the quality of peas remains to be further investigated.

#### 3.1.2. Milling

Milling utilizes mechanical force to break down particles into smaller pieces or fine particles [97], which can be used to make pea flour from peas for further processing or direct consumption. Different milling methods and grinding conditions can affect the chemical components and some physicochemical characteristics of pea flours. The quality of the flour is closely related to the use of various screen aperture sizes (SASs) and rotor speeds [98,99,100]. For instance, a reduction in the SAS of the ultra-centrifugal mill could cause a notable increase in starch damage of pea flours, and the pea flour produced with a 500 µm aperture screen possessed the most stable pasting and thermal properties among different sizes of aperture screens [98]. In addition, the ultra-centrifugal mill with a 500 µm SAS and 16,000 rpm rotor speed could maintain the aroma profile of the milled yellow pea flour without producing other beany-related volatiles [98]. Furthermore, hammer milling was also applied for the processing of yellow split peas, which were hammer-milled at two rotor speeds (34 and 102 m/s) and with nine mill screen apertures (0.84–9.53 mm). It was revealed that the median particle size of pea flours was the lowest while the viscosity was the highest when milling at a rotor speed of 102 m/s with a screen aperture of 0.84 mm [100]. Moreover, a previous study revealed that hammer- and disc-milling methods with different settings and screen sizes resulted in differences in the particle size and particle size distribution of field peas, thereby affecting their in vitro starch and protein digestion properties [101]. The hammer-milled field peas had higher hydration and digestion properties than those produced by the disc-mill, which may be due to the differences in their milling forces, frictional heat generation, and ability to degrade starch and protein molecules [101]. In addition, it was found that the protein yield of peas treated with dry milling increased from 59.5% to 67.1% [99].

#### 3.1.3. Soaking

Soaking is an important process that takes place before other food processing treatments such as cooking, microbial fermentation, and germination [102]. Soaking does not significantly alter the chemical compositions of pea flours but does have some impacts on their physicochemical properties [103]. Soaking could disrupt the protein and fibrous matrix around starch granules in yellow peas, making them swell to a high degree during gelatinization, thereby increasing the gelatinization viscosity of yellow pea flours [103]. In general, soaking could increase the protein solubility of yellow pea flours to some extent, which may be due to the breakdown of proteins by the proteolytic enzymes [103]. In addition, a previous study found that soaking could significantly reduce the contents of lectins and oxalates in peas, but had no impact on the content of phytic acid [9]. This result was reported in only one study, and the effect of soaking on phytic acid content remains to be verified. Soaking also significantly reduced the activity of enzyme inhibitors [104]. However, another study did not observe any reduction in trypsin-inhibitory activity of peas after soaking treatment [105]. More studies are needed to further clarify these results. Furthermore, the effect of soaking on other nutrients of peas has not been investigated.

#### 3.1.4. Cooking

The cooking quality is measured by the firmness and cooking loss of cooked beans, which can be determined by composition, nutritional, and physicochemical properties [27]. Cooking brings about several changes in the chemical composition of peas. A previous study found that the rapidly digestible starch content of peas increased with increasing cooking temperature, while the resistant starch content showed a decreasing trend [14]. In addition, the concentrations of Mg, Mn, Fe, Cu, and Zn in pea flours significantly decreased when using traditional cooking (i.e., boiling in water) [106]. Cooking also affected the contents of bioactive compounds in peas, such as reducing total polyphenols by 48–70%, reducing total saponins by 14–30%, and reducing total oligosaccharides by 20–44% [107]. However, on the other hand, cooking can improve the nutritional quality by inactivating or reducing the level of anti-nutritional factors [9]. In addition, cooking could increase free phenolic acids in peas, and reduce bound phenolic acids [108]. Furthermore, the content of β-carotene in peas increased significantly after cooking treatment, and the true retention coefficient of the cooked β-carotene content was 128.3% [109].

Some non-traditional cooking methods, such as pressure, microwave, and slow cooking, have also been used to process peas. Peas cooked by the slow cooking method (85 °C and 9 h) possessed higher levels of resistant starch (RS) fractions and lower levels of rapidly digestible starch (RDS) fractions than those cooked by the traditional boiling method [110]. On the other hand, the physical properties and chemical composition of peas play critical roles in influencing the cooking properties, such as the cooking time and texture [31]. For instance, the firmness of cooked peas was found to be significantly correlated with their contents of protein, starch, and ash, and it was also affected by the viscosity of pea flours and the hydration capacity of pea seeds [27]. However, knowledge is still limited regarding the effect of the physicochemical properties of pea flours on the cooking quality of peas.

### 3.2. Modification of Pea Starches

Native starch tends to produce a poor-textured, sticky, and rubbery paste when heated, and forms an unwanted gel when cooled. Accordingly, starch is often modified by physical, chemical, and enzymatic methods to enhance various functional properties [111,112]. Recently, many investigations have described that physicochemical characteristics and functional properties of pea starches can be improved by different techniques, such as heat-moisture treatment, ultrasound treatment, acidic and enzymatic treatments, γ-irradiation treatment, microwave treatment, annealing treatment, germination treatment, or their combinations, which are reviewed and discussed below. RS has gradually become a popular food component due to its beneficial effects and heat resistance during food processing. To improve the RS content, heat-moisture treatment (HMT) combined with malic acid treatment was applied for the modification of pea starch [113]. It was found that the application of HMT before the esterification reaction by malic acid could improve the degree of substitution of the starch products. The possible reason was that HMT could raise the availability of the starch granules and make it easy for malic acid to penetrate the starch granules, resulting in the dramatic increase in the RS and SDS contents of pea starch and the decrease in its enzymatic sensitivity [113]. Accordingly, the high content of RS and the low digestibility of the modified starch may be used to produce low-calorie foods and health products.

Ultrasound has obtained much attention as a green technology for the modification of food components as it can be simple, reliable, and environmentally friendly. Recently, ultrasound treatment (UT) and heat-moisture treatment (HMT) were utilized for the modification of the physicochemical characteristics of pea starches [114]. It was found that these treatments could reduce the crystallinity, molecular mass, swelling power, and solubility of pea starches at 70–90 °C. Furthermore, the UT method remarkably could promote the content of apparent amylose in pea starches from 34.08% to 37.82%. Nevertheless, the content of amylose significantly reduced after HMT treatment and dual treatment with HMT and UT compared to non-treated starches [114]. On the other hand, all modifications increased the content of RS, and the HMT treatment and dual treatment with HMT and UT reduced the RDS in pea starches, indicating that the digestive ability of the modified starch was reduced [114]. Furthermore, it was found that the UT-assisted HMT treatment could improve the viscosity and high-temperature stability of the starch paste compared to others. In addition, γ-irradiation is another non-conventional method for the modification of starch, which is fast, simple, and environmentally friendly. Recently, ultrasound treatment followed by γ-irradiation was employed to modify the physicochemical properties of pea starches [115]. It was found that no significant difference was observed in pea starches after only ultrasound treatment, while large differences were found in the physicochemical and functional properties of pea starches upon dual treatment with ultrasound and γ-irradiation [115]. The apparent amylose content of pea starches significantly decreased after dual treatment with ultrasound and γ-irradiation. With the dual treatment of pea starches by ultrasound and γ-irradiation, their solubility, water/oil absorption capacity, and transmittance significantly increased, while their swelling index, pasting properties, and syneresis decreased [115]. The improved physicochemical and functional properties of pea starches following the treatment with ultrasound and γ-irradiation may be beneficial to expanding related food applications. Furthermore, in order to obtain desirable starch materials for the application in the food industry, a recent study systematically evaluated the impacts of different treatments on the physicochemical and functional properties of pea starches, including autoclaving (ACP), microwave cooking (MCP), autoclaving with ultrasound (UP), autoclaving with acid hydrolysis (AHP), and autoclaving with pullulanase debranching (PDP) [116]. It was found that the typical C-type crystal form of the original starch was transformed into a B-type crystal structure upon different treatments, and the semi-crystalline layer structure also disappeared after different processing treatments. In addition, the long-range crystallinity of pea starches was decreased, while the short-range crystallinity was relatively increased upon different treatments [116]. The findings in this study also showed that MCP was less effective than other treatments for the improvement of the formation of stabilized double helical structure, and ACP had a less pronounced effect for the promotion of the formation of short-range ordered structure than that of UP, AHP, and PDP.

Annealing is one of the most important physical processing treatments to improve the physicochemical properties of starch [117]. A rapid ethanol-assisted annealing method was developed and applied for the modification of pea starches [117]. It was found that the peak viscosity of the modified pea starch decreased, while its trough and final viscosities notably increased. In addition, its crystalline structure was changed from a C-type to an A-type. Its pasting temperature was also increased by ethanol-assisted annealing treatment, while its enthalpy change value decreased. In addition, after the pea starch was annealed in 60% of ethanol solution for 3 h, its gelatinization temperature was 80.97 °C, which was higher than that of the original starch. Furthermore, the annealed starch exhibited a significant reduction in swelling and solubility [117]. The ethanol-assisted annealing method not only shortens the incubation time for annealing, but also improves the thermal stability, shear stability, and acid stability of the pea starch. Nonetheless, the related mechanism of action of ethanol solution annealing requires further investigation. A novel annealing method, annealing with plasma-activated water (PAW-ANN), was applied for the modification of the physicochemical properties of pea starches [118]. It was found that the PAW-ANN treatment could improve the long and short-range ordered structure of the pea starch, increase its gelatinization enthalpy, reduce its peak viscosity, and promote its gel strength [118]. Furthermore, the plasma-activated water (PAW) from plasma treatment of distilled water has the advantages of uniform function, being green, and environmental protection.

Germination is a bioprocess where enzymes are released and activated, and several investigations have revealed that germination treatment can affect the physicochemical and functional properties of starch [6]. Recently, the effects of germination on the structures and physicochemical characteristics of pea starches was studied [6]. It was revealed that the germination treatment could significantly promote the amylose content and particle size distribution of pea starches, but slightly reduced the relative crystallinity [6]. On the other hand, the germination treatment could significantly reduce the peak viscosity, valley viscosity, final viscosity, and gelatinization enthalpy of pea starches [6]. Collectively, germination treatment can significantly affect the structures and physicochemical characteristics of pea starches, which may provide a theoretical basis for promoting the application of germinated pea starches in the food industry. Nevertheless, although various methods have been applied for enhancing the physicochemical and functional properties of pea starch, the exact underlying mechanisms are still unclear and deserve further exploration.

### 3.3. Modification of Pea Proteins

The pea protein and its hydrolysates exhibit various beneficial effects, such as antioxidant effects, anti-hypertensive effects, and regulating gut microbiota, as well as different functional properties, such as solubility, water-binding effect, emulsifying, and gelling properties. Nevertheless, the application of pea protein in food systems is still limited owing to its poor functional properties [17]. A recent review summarized the physical, chemical, and enzymatic modification methods for improving the bioactivities and functionalities of pea proteins [17]. Advances in several enzymatic and physical modification methods for promoting functional characteristics of pea proteins are introduced below.

A recent study systematically evaluated enzymatic hydrolysis to improve the functional properties and sensory properties of pea protein isolates as well as to reduce the potential allergens. Eleven proteolytic enzymes at different hydrolysis times (15, 30, 60, and 120 min) were applied for the modification of pea protein isolates [119]. It was found that most enzymes could promote the functional properties of pea protein isolates, especially the solubility at pH 4.5 and the foaming ability. Furthermore, their bitterness changed obviously after enzymatic hydrolysis, which was strongly correlated with the degree of hydrolysis. On the other hand, the degradation of Pis s 1 and Pis s 2 indicated that enzymatic hydrolysis could be a potential method to reduce the main pea allergens [119]. Furthermore, a novel method, supercritical carbon dioxide + ethanol extraction (SCD-EA), was applied for improving the techno-functionalities of pea protein isolates. It was found that SCD-EA could notably increase its solubility, emulsion, and foaming properties, which can make them more suitable for food applications [120]. Moreover, an efficient ultrasound-assisted alkali extraction method (UAAE) was developed to obtain a high level of pea protein isolates and its effect on protein functional properties and biological effects was also evaluated [121]. Ultrasound treatment could affect the secondary and tertiary structures of pea protein isolates, and significantly promote related functional properties, such as solubility, water/oil binding ability, foaming/emulsifying ability, and gel formation ability. On the other hand, ultrasound treatment could increase the in vitro antioxidant activity and angiotensin-converting enzyme inhibitory activity of pea protein isolates.

Micro-fluidization is a unique high-pressure homogenization technique, which can modify the structure of macromolecules leading to improved physicochemical and functional properties [122]. Industrial-scale micro-fluidization (ISM) was applied to modify pea proteins [123]. It was found that the ISM treatment could effectively improve the solubility of pea proteins. Indeed, the enhanced solubility of pea proteins was embodied in the increased turbidity, reduced particle sizes, increased specific surface area, and changed morphology from thick blocks to thin fragments [123]. ISM treatment could promote the transformation of large insoluble proteins or aggregates into soluble particles by breaking disulfide bonds. In addition, micro-fluidization could be used to modulate the emulsifying properties of pea globulins depending on their initial denaturation state [124]. Collectively, these findings suggest that micro-fluidization treatment may be a potential method to improve the functional properties of pea proteins.

## 4. Health Benefits of Pea and Its Components

**Figure 2:**
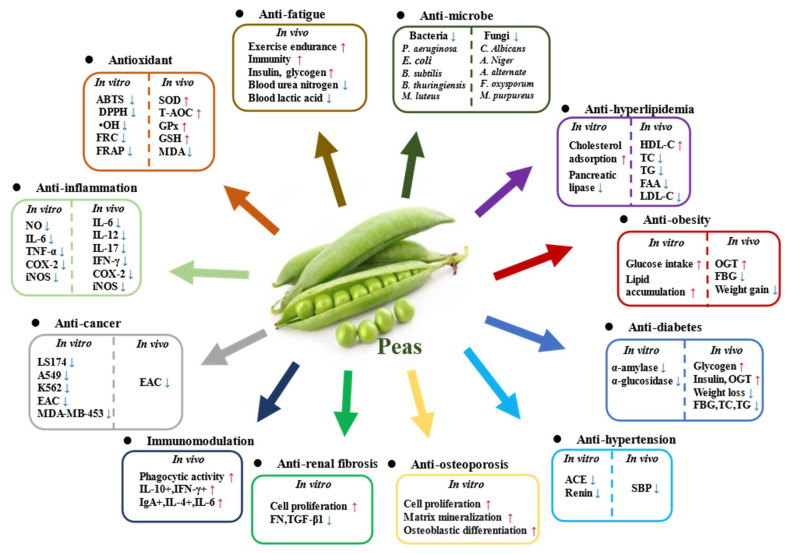
Biological activities of pea and its bioactive components. Pea and its bioactive components possess various health benefits in vitro and in vivo, such as antioxidant, anti-inflammatory, immunomodulatory, anti-cancer, anti-hypertensive, anti-obesity, anti-diabetic, anti-hyperlipidemia, anti-fatigue, antimicrobial, anti-osteoporosis, and anti-renal fibrosis effects. A594, lung carcinoma; ABTS, 2,2 azinobis 3-ethylbenzo-thiozoline-6-sulfonic acid; ACE, angiotensin I-converting enzyme; COX-2, cyclooxygenase-2; DPPH, 2,2-Diphenyl-1-picrylhydrazyl; EAC, Ehrlich ascites carcinoma; FAA, free amino acid; FBG, fasting blood glucose; FN, fibronectin; FRAP, ferric reducing antioxidant power; FRC, ferric ion-reducing capacity; GPx, glutathione peroxidase; GSH, glutathione; HDL-C, high-density lipoprotein cholesterol; IFN-γ, interferon-gamma; IgA+, immunoglobulin class A+; IL, interleukin; iNOS, inducible nitric oxide synthase; K562, myelogenous leukemia; LS174, human colon denocarcinoma; LDL-C, low-density lipoprotein cholesterol; MDA, malondialdehyde; MDA-MB-453, breast carcinoma; NO, nitric oxide; OGT, oral glucose tolerance; ·OH, hydroxyl radical; SBP, systolic blood pressure; SOD, superoxide dismutase; T-AOC, total antioxidant capacity; TC, total cholesterol; TG, triglyceride; TGF-β, transforming growth factor beta; TNF-α, tumor necrosis factor α.

The various health benefits of peas, such as antioxidant, anti-inflammatory, anti-hypertensive, anti-obesity, anti-cancer, anti-fatigue, anti-diabetic, antimicrobial, and anti-renal fibrosis effects, have been demonstrated in different in vitro and in vivo experimental studies (Figure 2). Table 2 summarizes the sample types, experimental models, and main results of the health benefits of pea and its components. Most of the samples used in these studies were prepared from pea seeds and their by-products, such as pea seed coats and pea hulls. There are also many studies focusing on the biological activities of pea components, such as polyphenols, dietary fiber, proteins, and peptides. In addition, few studies have focused on pea glycoproteins, protease inhibitors, and lectins. The main biological activities of pea and its components are discussed below.

### 4.1. Antioxidant Activity

Many studies have revealed that pea and its by-products possess remarkable antioxidant activity due to the existence of various bioactive components (e.g., polyphenols, polysaccharides, and peptides) [21,22,128]. Among them, seeds and seed coats represent the most frequently studied fractions for their TPC or free radical scavenging properties, with a combination of popular in vitro assays using diverse mechanisms, including 2,2′-azino-bis (3-ethylbenzothiazoline-6-sulphonic acid) (ABTS), 2,2-Diphenyl-1-picrylhydrazyl (DHHP), ferric reducing antioxidant power (FRAP), ferric ion-reducing capacity (FRC), ferrous ion-chelating capacity (FCC), and lipid peroxidation models [65,68,82,126,127]. In general, a higher level of TPC is aways associated with a stronger antioxidant capacity [69,82,125,126]. For instance, the dark-colored (e.g., brown and dark purple) pea seeds, which contained more TPC and TFC than light-colored seeds, showed better antioxidant activity [68,125,126]. The high TPC of the darker varieties exhibited stronger antioxidant activity, which could be related to the existence of gallic acid, epigallocatechin, naringenin, and apigenin [126]. In addition, different extraction conditions and processing techniques can also affect the phenolic content and antioxidant activity. For example, the antioxidant activity of ethyl acetate extract was found to be the highest compared to methanolic and aqueous extracts, which might be owing to its higher level of TFC [127]. In addition, microwave drying could affect the bioactive components and antioxidant capacity of green peas [74]. Interestingly, peas dried at a microwave power of 100 W had the highest TPC and antioxidant activity among all tested conditions [74]. Furthermore, the germination treatment could also result in changes in the amount and types of phenolic compounds in peas, improving the antioxidant activity. For example, germination caused an increase in the gallic acid and syringic acid contents in pea sprouts. Indeed, vanillin and ferulic acid could also be found in pea sprouts with increasing germination time [70]. Moreover, the effect of in vitro digestion on the level of TPC and antioxidant capacity of pea hulls was studied [69]. It was found that there was no significant increase in individual phenolics after in vitro digestion, suggesting that in vitro digestion had only a small effect on the phenolics released [69]. Nevertheless, the in vitro digestion model only simulates the enzymes and pH of the digestive system, which needs to be further clarified by corresponding in vivo studies.

Furthermore, cellular experiments demonstrated that treatment with different doses of ethanolic extract from pea flours could restore the intracellular lipid, malondialdehyde, and antioxidant enzyme levels in oleic acid-induced HepG2 cells [65]. In animal studies, the daily administration of pea flours to rats induced by a high-fat diet could improve the antioxidant capacity, as evidenced by the increased activities of serum glutathione peroxidase (GSH-Px), superoxide dismutase (SOD), and total antioxidant capacity (T-AOC), and the reduced level of serum malondialdehyde (MDA) [65]. In addition, both methanolic extracts from green pea hulls and yellow pea hulls could improve the GSH-Px, SOD, and T-AOC activities in the plasma, and reduce the content of MDA [73,130]. Several studies have also investigated the bioavailability, metabolic characteristics, and antioxidant activities of pea hull polyphenols in animal models [73,130]. It was demonstrated that the main phenolics in pea hulls, especially catechin, pentaphyllin, kaempferol, quercetin and their derivatives, could be transformed during gastrointestinal digestion and absorbed in their native or metabolite form, exerting antioxidant effects in the body. Furthermore polyphenols, polysaccharides, and peptides also exhibited significant antioxidant activities [128,129]. At present, the antioxidant activity of peas has mainly been evaluated in vitro and in vivo, whether pea can fight against oxidative stress in humans remains unknown and well-designed clinical trials should be designed to verify it.

### 4.2. Anti-Inflammatory Effect

Pea and its components possess remarkable anti-inflammatory effects. For instance, a recent study showed that polyphenols released from green pea hulls during in vitro digestion could alleviate the LPS-induced inflammation in Caco-2/RAW 264.7 coculture cell models [131]. Pea hull polyphenols could inhibit the release of nitric oxide (NO), interleukin-6 (IL-6), and tumor necrosis factor α (TNF-α) from Caco-2/RAW 264.7 coculture cells, as well as inhibiting the mRNA expression of cyclooxygenase-2 (COX-2) and inducible nitric oxide synthase (iNOS). In addition, pea protein hydrolysates from yellow field pea seeds also exhibited a remarkable in vitro anti-inflammatory effect, which could inhibit the secretion of NO, IL-6, and TNF-α from LPS/IFN-γ-activated RAW 264.7 macrophages [132].

In animal studies, the supplementation of green pea flours could ameliorate the severity of dextran sulfate sodium (DSS)-induced colitis in C57BL/6J female mice, which was associated with the suppression of inflammation, mucin depletion, and endoplasmic reticulum stress in the colon [133]. In addition, polyphenols extracted from the green pea hulls could also ameliorate colitis in C57BL/6 male mice by activating the Kelch-like ECH-associated protein 1 (Keap1)-NF-E2-related factor 2 (Nrf2)-antioxidant responsive element (ARE) signaling pathway, regulating gut microbiota, and increasing the levels of short-chain fatty acids (SCFAs). Indeed, quercetin, kaempferol, catechin, and their derivatives were identified as the main compounds in the polyphenolic extract of green pea hulls by UHPLC-LTQ-MS [77]. Furthermore, the albumin extracts from pea seeds showed anti-inflammatory effects in DSS-induced colitis in C57BL/6J male mice [134]. It was found that two pea seed albumin extracts, including pea seed extract (PSE) containing an albumin fraction and non-starch polysaccharide fraction, and the albumin fraction from PSE, could reduce microscopic histological damage in comparison with untreated colitis mice, and ameliorate the colonic mRNA expression of different pro-inflammatory markers. Collectively, these studies demonstrated that pea flours, polyphenols, proteins, and non-starch polysaccharides exhibited remarkable anti-inflammatory effects.

### 4.3. Regulation of Metabolic Syndrome

**Figure 3:**
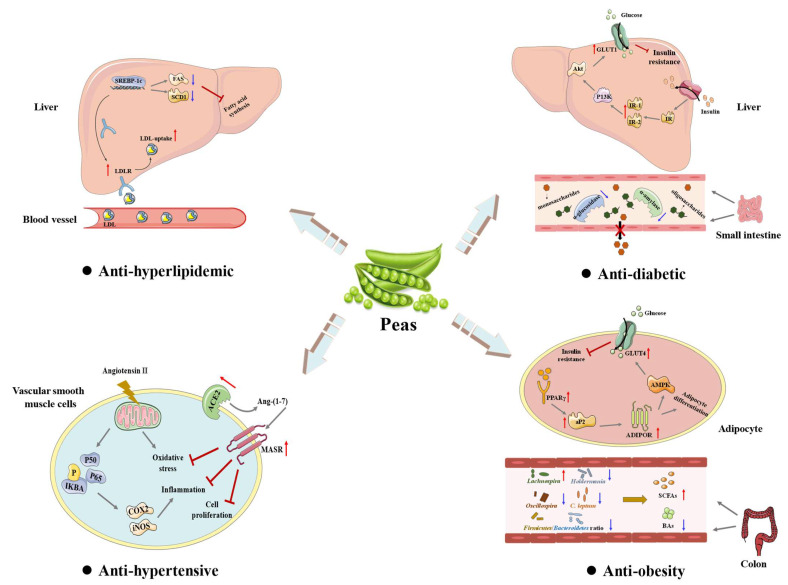
The potential mechanisms of pea and its bioactive components on the regulation of metabolic disorders. (1) Pea and its bioactive ingredients have anti-hyperlipidemic effects by activating the SREBP2 pathway, upregulating LDLR expression, promoting serum LDL-C clearance, and downregulating FAS and SCD expression to inhibit fatty acid synthesis. (2) Pea and its bioactive components have anti-diabetic effects by activating the expression of GLUT1 to promote glucose absorption, upregulating the expression of the insulin receptor substrates IRS-1 and IRS-2 to reduce insulin resistance, as well as inhibiting the activity of small intestinal alpha-glucosidase and alpha-amylase and inhibiting the breakdown of dietary polysaccharides into easily absorbed oligosaccharides and monosaccharides. (3) Pea and its bioactive components have antihypertensive effects by upregulating ACE2 and MASR expression through the ACE2-Ang-(1-7)-MASR axis to improve angiotensin II-induced superoxide production, inflammation, and proliferation in vascular smooth muscle cells. (4) Pea and its bioactive components have anti-obesity effects by inducing adiponectin and insulin-responsive GLUT4 to stimulate glucose uptake and improve insulin resistance, upregulating PPARγ and aP2 expression levels to stimulate adipocyte differentiation, as well as by modestly altering the microbial status of the gut, such as downregulation of the Firmicutes/Bacteroidetes ratio, leading to changes in SCFAs and Bas. ACE, angiotensin I-converting enzyme; ADIPOR, adiponectin receptor; AMPK, adenosine 5′-monophosphate (AMP)-activated protein kinase; Ang, angiotensin; Akt, protein kinase B; aP2, adipocyte fatty acid-binding protein; BAs, bile acids; COX-2, cyclooxygenase-2; FAS, fatty acid synthase; GLUT, glucose transporter; iNOS, inducible nitric oxide synthase; IKBA, inhibitory kBa; IRS, insulin receptor substrate; LDL, low-density lipoprotein; LDLR, low-density lipoprotein receptor; MASR, Mas receptor; P13K, phosphatidylinositol 3-kinase; PPARγ, peroxisome proliferator-activated receptor γ; SCD, stearoyl coenzyme A desaturase; SCFAs, short-chain fatty acids; SREBP, sterol regulatory element-binding protein.

Metabolic syndrome (MS) is a group of clinical syndromes characterized by hypertension, dyslipidemia, obesity, and hyperglycemia. Many in vitro and in vivo experimental results have shown that pea and its components are effective in regulating various metabolic disorders (Figure 3).

Peptides derived from pea protein hydrolysates were found to possess remarkable anti-hypertensive effects, as evidenced by inhibiting enzymatic activities of angiotensin-converting enzyme (ACE) and renin [28,135,138]. In addition, a tripeptide LRW (Leu-Arg-Trp) derived from the pea protein legumin was found to exhibit anti-hypertensive activity by reducing angiotensin II-induced superoxide production, inflammation, and proliferation in vascular smooth muscle cells. The anti-hypertensive activity of LRW appeared to involve the improvement of the ACE2-Ang-(1-7)-MasR axis and modulation of the nuclear factor-ĸB (NF-ĸB) pathway [137]. Furthermore, animal studies also showed that pea protein hydrolysates exhibited potential anti-hypertensive properties [28,138]. For instance, the long-term oral administration of pea protein hydrolysates (1% casein substitute in the diet) in spontaneously hypertensive rats could decrease the systolic blood pressure by a maximum of −36 mmHg after 3 weeks [138]. Collectively, these results suggest that pea and pea protein hydrolysates can be developed into health products for the prevention of hypertension.

Pea and its bioactive components also exhibit hypolipidemic effects in vitro and in vivo. It was found that the daily intake of peas could significantly restore the levels of serum total cholesterol (TC), low-density lipoprotein cholesterol (LDL-C), and high-density lipoprotein cholesterol (HDL-C), as well as alleviate liver lesions in rats fed a high-fat diet [65]. In addition, the effect of autoclaved extract (AE) of pea pods (mainly polyphenols and dietary fiber) on lipid profiles in rats with high-sucrose diet-induced hyperlipidemia was discussed [139]. It was found that AE could remarkably reduce the serum triglyceride (TG) and TC levels in rats fed a high-sucrose diet. Moreover, AE could significantly promote the growth of *Bifidobacteria* in the cecum. Furthermore, studies showed that pea protein isolates caused a significant reduction in serum TC and TG levels in rats, which appeared to influence the cellular lipid homeostasis by improving the hepatic cholesterol uptake genes and decreasing the fatty acid synthesis genes [140]. Collectively, peas can be developed into health products for the prevention of hyperlipidemia.

Obesity is usually a serious, obvious, but also the most neglected global health issue. Promoting adipocyte differentiation can be effective in ameliorating the metabolic disorders of obesity. Recently, the effect of pea protein hydrolysates on adipocyte differentiation has been investigated by using 3T3-L1 murine pre-adipocytes [141]. It was found that the pea vicilin hydrolysate (PVH) could promote the mRNA expression of the adipocyte fatty acid-binding protein and reduce that of preadipocyte factor-1, thereby promoting adipocyte differentiation. In addition, PVH could induce the expression of adiponectin and insulin-responsive glucose transporter 4 and stimulate the uptake of glucose. The level of peroxisome proliferator-activated receptor gamma (PPARγ) was upregulated during adipocyte differentiation. Collectively, this study indicated that PVH could stimulate adipocyte differentiation by partially upregulating PPARγ expression and its ligand activity [141] (Figure 3). Furthermore, pea proteins and their related hydrolysates, and pea dietary fiber also exhibited beneficial effects on obesity. It was found that pea seed flours could attenuate weight gain in diet-induced obese rats, and pea dietary fiber and pea flours could remarkably decrease the final percent body fat compared with the control [55]. On the other hand, pea dietary fiber could reduce the Firmicutes/Bacteroidetes ratio, and decrease the abundance of *Clostridium leptum*, which was increased in obese individuals. Furthermore, to understand the impacts of pea dietary fiber on the regulation of the microbiota–host metabolic axis in obesity, a 12-week clinic trial with 53 adults who were overweight or obese was carried out [51]. It was found that the supplementation of the diet with pea fiber could prevent detrimental changes in the metabolic profile, which may be owing to a modest change in the gut microbial profile, resulting in alterations in short-chain fatty acids (SCFAs), bile acids (BAs), and ketone bodies key signaling molecules linked to obesity [51].

Diabetes is becoming one of the most critical public health challenges and economic burdens in the 21st century. As shown in Table 2, pea proteins, pea protein hydrolysates, pea glycoproteins, and pea dietary fiber exhibited remarkable anti-diabetic activity in vitro and in vivo. Both pea protein hydrolysates and pea glycoproteins could inhibit the enzymatic activities of digestive enzymes (e.g., α-amylase and α-glucosidase) [142,143]. In addition, the mechanism of pea glycoprotein (PGP2)-induced blood glucose reduction was investigated using streptozotocin (STZ)-induced diabetic mice [144]. It was found that PGP2 could lower diabetic weight loss, fasting blood glucose level, serum levels of total cholesterol, triglycerides, and low-density lipoproteins, while promote the oral glucose tolerance level and secretion of insulin. Furthermore, PGP2 could promote the expression of insulin receptor substrates IRS-1 and IRS-2 as well as glucose transporter 1 (GLUT1) in liver tissue, suggesting that it could regulate blood glucose according to the IRS-PI3K-Akt-GLUT1 signaling pathway [144] (Figure 3). Furthermore, the hypoglycemic activity of pea oligopeptides in HFD- and STZ-induced type 2 diabetes (T2D) mice has been investigated [145]. It was found that pea oligopeptides could decrease the level of blood glucose, while improving the glucose tolerance, promoting the release of insulin and the synthesis of glycogen, and protecting liver and kidney tissues in diabetic mice [145]. Moreover, animal studies also revealed that pea dietary fiber exhibited potential hypoglycemic effects [50,55], which could obviously reduce the serum glucose level and promote the oral glucose tolerance level. Furthermore, a clinical trial was carried out to investigate the effect of pea protein on postprandial glycemic, insulinemia, glucose-dependent insulinotropic polypeptide (GIP), and glucagon-like peptide-1 (GLP-1) response following a high-carbohydrate beverage intake in forty-five healthy male and female adults aged from 19 to 55 years old [146]. It was found that the intake of pea protein could decrease postprandial glycaemia and stimulate the production of insulin in healthy individuals. However, there was no clear difference in GIP and GLP-1 release between the control and pea protein drinks. Collectively, these results indicate that pea and its components possess potential applications in the prevention and management of diabetes.

### 4.4. Antimicrobial Effect

Several studies have shown that pea and its by-products possess remarkable antimicrobial activities, such as pea peptides [147], pea lectins [148], polyphenols derived from pea peels [127], and soluble polysaccharides derived from pea pods [149]. It was found that 11S pea globulin possessed obvious inhibitory effects against Gram-positive and Gram-negative bacteria with the minimum inhibitory concentrations (MICs) ranging from 120 to 160 and 145 to 190 μg/mL, respectively [147]. On the other hand, the 11S pea globulin could also notably inhibit the growth of several fungi with the MICs ranging from 55 to 80 μg/mL. In addition, pea lectins could inhibit the growth of several bacteria (such as *Klebsiella pneumonia*, *Pseudomonas aeruginosa*, and *Staphylococcus aureus*), with the MICs ranging from 62.5 to 125 μg/mL [148]. Furthermore, soluble polysaccharides derived from pea pods possessed obvious inhibitory effects against Gram-negative and Gram-positive bacteria at the concentration of 50 mg/mL [149]. Moreover, it was found that the ethyl acetate extract of pea peels possessed stronger inhibitory effects against several microorganisms (e.g., *Staphylococcus aureus*, *Salmonella enterica*, *Escherichia coli*, *Pseudomonas aeruginosa*, *Aspergillus niger*, and *Candida albicans*) than the methanolic and water extracts. In particular, the water extract of pea peels exhibited no inhibitory effects against these microorganisms [127]. Finally, these studies indicate that pea and its components exhibit potential antimicrobial activity and can be potentially used as antimicrobial agents in the food industry.

### 4.5. Anti-Renal Fibrosis Effect

Renal fibrosis usually results in glomerulosclerosis and interstitial fibrosis, which can progressively develop into long-term kidney disease [155]. The protein hydrolysate of green peas degraded by bromelain (PHGPB) was found to promote the proliferation of SV40 MES 13 mesangial cell induced by high glucose levels [151]. On the other hand, PHGPB could reduce the levels of fibronectin (FN) and transforming growth factor-β 1 (TGF-β1) in mesangial cell lines of diabetic glomerulosclerosis, exhibiting great potential for antifibrosis in chronic kidney disease [151]. In addition, a previous study further revealed the role of PHGPB in TGF/SMAD signaling to prevent renal fibrosis [150]. It was found that PHGPB could decrease the level of TGF-β1 in high-glucose-induced SV40 MES 13 cells. In addition, the provision of PHGPB could inhibit the gene expression of *SMAD 2, SMAD 3*, and *SMAD 4* genes, but increase the gene expression of *SMAD 7* [150]. Collectively, peas have shown certain therapeutic effects in the treatment of renal fibrosis, but the relevant studies are not yet complete, and it is important to fill the gaps in clinical studies in the future.

### 4.6. Other Beneficial Effects

Pea and its bioactive components also have other beneficial effects. For instance, the bioactive tripeptide LRW (Leu-Arg-Trp) obtained from pea protein could positively regulate the activity of osteoblasts via the Akt/Runx2 pathway, suggesting its potential application for the prevention of osteoporosis [153]. In addition, water extracts from pea seed coats showed cytotoxicity against various cancer cell lines, such as LS174, MDA-MB-453, A594, and K562 [126]. Meanwhile, lectins from pea seeds also exhibited obvious inhibitory effects against Ehrlich ascites carcinoma cells in vitro and in vivo by arresting the cell cycle at the G2/M phase [152]. Furthermore, it was found that peptides derived from pea protein hydrolysates exhibited immunomodulatory effects in vivo, which could increase the phagocytic activity of macrophages and stimulate the gut mucosa immune response as well as enhance the production of IL-6 via the simulation of toll-like receptor-2 (TLR2) and TLR4 [132]. Moreover, the anti-fatigue effect of pea peptides was studied. It was found that pea peptides could enhance the glycogen content in the muscle and liver, eliminate accumulated lactic acid, inhibit oxidation in the body caused by free radicals, and improve the immune function of the body [154].

## 5. Applications of Pea and Its Components

**Figure 4:**
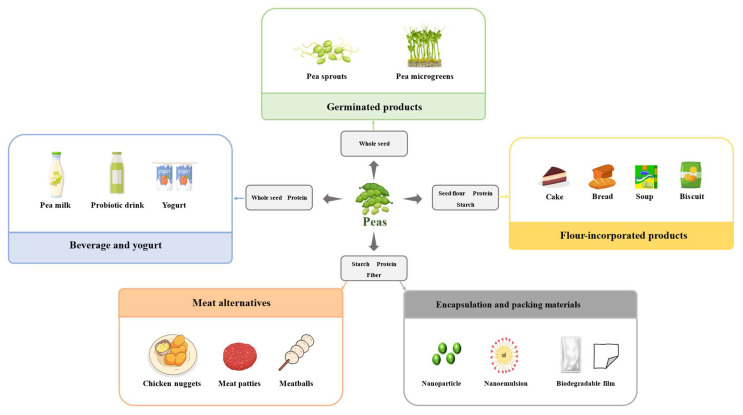
Food-related applications of pea and its components. Pea and its main components are widely applied in the food system, such as pea beverages and yoghurts, germinated pea products, pea flour-incorporated products, pea-based meat alternatives, and pea-based encapsulation and packing materials.

A recent review has summarized various applications of pea protein in the food industry, which focuses on the pea-protein-based encapsulation systems for bioactive compounds, pea-protein-based films, pea-protein-based extruded products, and pea-protein-based flour products, as well as pea protein being used as an alternative for animal products [17]. Therefore, this section updates the most recent applications of pea and its main components in the food system, such as pea beverages and yoghurts, germinated pea products, pea flour-incorporated products, pea-based meat alternatives, and encapsulation and packing materials (Figure 4).

### 5.1. Pea Beverages and Yoghurts

Plant-based milk has become very popular in the world due to its high nutritional and health values. Pea seeds can be made into pea milk by directly soaking and mixing with fresh water. Nevertheless, uncomfortable sensory characteristics such as “grassy” and “beany”, which result from hexanal, nonanal, and hexanol, can be easily generated during the processing of pea milk [156,157,158]. These uncomfortable sensory characteristics notably restrict the application of peas in protein beverages or plant-based yoghurts. Recently, to decrease the contents of “off-flavor” components during the processing of pea milk, a comparative study examined the effects of different lipoxygenase (LOX) inhibitors, modulators, and high hydrostatic pressure on the flavor and sensory characteristics of pea milk [158]. It was found that the addition of inhibitors (e.g., ascorbic acid, quercetin, epigallocatechin-3-gallate, and reduced glutathione) could affect the enzymatic activity of LOX and change the contents of α-linolenic acid and linoleic acid in pea milk, thereby reducing the contents of hexanal and 2-pentylfuran [158]. In addition, high hydrostatic pressure treatment could also significantly reduce the content of hexanal. Furthermore, modulators (e.g., pea proteins, sodium sulfate, and propylene glycol) could strengthen the interactions between flavor compounds and pea proteins, thereby reducing the production of hexanal and hexanol [158]. On the other hand, the sensory profiles of pea milk showed a trend towards a reduction in “fatty” intensity and an increase in that of “milk-like” after the different treatments [158]. Moreover, a recent study also indicated that different pre-treatments for pea milk making, including alkali water soaking at 25 °C (Alk), dry dehulling and alkali water soaking (Deh + Alk), boiling water blanching, wet dehulling and alkali water soaking (Bla + Alk), as well as boiling water blanching, dehulling, and acid water soaking (Bla + Acid), remarkably affected the contents of volatile compounds in pea milk [159]. More specifically, Bla + Alk and Bla + Acid treatments showed lower contents of total volatiles than in Alk and Deh + Alk treatments, suggesting that the blanching played a critical role in the decrease in the volatiles content through inactivating LOX. Furthermore, ultra-high temperature treatment could change the aroma profile of pea protein beverages [157]. Lipid oxidation and the Maillard reaction were identified as the two main aroma formation pathways that occur during the ultra-high temperature treatment, which could cause the increase in compounds with plastic, nutty, and dusty notes in pea protein beverages [157]. Moreover, a probiotic beverage enriched with pea and rice proteins was developed [160]. It was found that the quality of pea and rice proteins was similar to that of casein after lactic acid bacteria fermentation. More specifically, lactic acid bacteria fermentation could significantly increase the protein efficiency ratio (PER) and the net protein ratio (NPR) from 1.88 to 2.32 and 1.66 to 2.30, respectively, thereby improving the protein quality of probiotic beverages [160].

Plant-based yoghurts, or dairy-free yoghurts are obtaining increasing acceptance in the world. Pea milk can be easily turned into pea yogurts by lactic acid bacteria fermentation [161]. In order to make pea-protein-based products similar to a conventional yoghurt, five mixtures of milk and pea proteins were fermented by ten starter cultures of lactic acid bacteria. It was found that the increase in pea concentration could lead to products with higher acidity, higher syneresis, and lower firmness. Furthermore, considering both the sensory characteristics and fermentation parameters, starter cultures (e.g., *Streptococcus thermophilus* + *Lactobacillus delbrueckii*, *S. thermophilus* + *L. acidophilus*, and *S. thermophilus* + *L. casei*) possess good potential for the production of yoghurts with pea proteins [161]. Nevertheless, to enhance the firmness of these fermented yoghurts, further studies remain to be performed. Furthermore, the effect of pea milk preparation methods (e.g., Alk, Deh + Alk, Bla + Alk, and Bla + Acid) on the quality of non-dairy yoghurts was evaluated [159]. It was found that the gel hardness of pea yogurts prepared by Alk, Deh + Alk, and Bla + Alk was significantly improved, while the flavor of pea yogurt prepared by Bla + Acid was better than that prepared by other methods [159]. On the other hand, these different pre-treatments reduced the yield of acid formation during lactic acid bacteria fermentation of pea milk [159]. In addition, plant-based yogurts tend to be lower in protein than dairy products, and the long fermentation time can cause some texture issues. To overcome these challenges, the high-pressure processing (HPP) of plant protein ingredients possessed the potential to address these issues [162]. It was found that the strength of HPP-formed viscoelastic gels from pea proteins was comparable to commercial dairy yogurts. Interestingly, its gel strength also increased with the incorporation of oil [162]. These results provide a theoretical basis for developing new processes to produce plant-based yogurts. Nevertheless, to meet the nutritional needs of humans, pea beverage fortification can be achieved by enriching the nutrients lost during processing [163], which still needs to be studied in the future.

### 5.2. Germinated Pea Products

Peas can be used in a less processed form, such as pea sprouts and microgreens. Germination can be widely applied to edible seeds to improve their phytochemical compositions and biological functions [164]. Pea sprouts are rich in minerals and polyphenolics [70,165]. The sprouting can activate the enzymatic activity of phytase, which degrades phytate, thereby improving the bioaccessibility and bioavailability of minerals [166]. Furthermore, the germination process also has a significant effect on reducing flatulence-related oligosaccharides [165]. In addition, the COVID-19 pandemic has caused unprecedented disruption of food systems, leading to food shortages across supply chains, and underscoring the significance of food self-sufficiency [12]. Therefore, vegetables in the form of sprouts and microgreens may temporarily solve domestic food shortages caused by extreme weather, natural disasters, or public health emergencies such as the COVID-19 pandemic.

The germination strategy of pea seeds can be achieved by a few simple steps, mainly including sterilization, soaking, and germination [164]. Cold plasma (CP) is an emerging technology, and its application has also been extended to adjust seed germination performance [167]. A previous study showed that the proper cold atmospheric pressure plasma (CAPP) treatment had significantly positive effects on pea seed germination [168]. Furthermore, sprouts are commonly consumed as raw materials due to their nutritional value, but can be associated with infections from a range of foodborne pathogens [169]. Therefore, to inhibit foodborne pathogens, sterilization is mostly performed before seed soaking. Slightly acidic electrolyzed water (SAEW, pH 5.0–6.5) is produced by the electrolysis of hydrochloric acid with or without sodium chlorite, which has been widely utilized for inactivating or eliminating various foodborne pathogens on fruits and vegetables [169]. In a previous study, SAEW was applied for the production of pea sprouts [169]. It was found that the number of natural microbes was notably reduced by the SAEW treatment. In addition, the fresh weight, length, and edible rate of pea sprouts were remarkably improved by SAEW treatment. On the other hand, SAEW showed no adverse effects on fresh weight, length, soluble sugar, total protein, vitamin C, or flavonoids. Collectively, SAEW can be a promising sterilization method for the production of pea sprouts.

Fortifying pea sprouts with essential elements is one of the efficacious ways to promote their nutritional value. Previous studies developed pea sprouts rich in selenium, iodine, and zinc [170,171]. The method of soaking pea seeds with iodine and selenium solution successfully enriched the pea sprouts with the two elements; it also changed some morphological and physiological parameters of pea sprouts [170]. Se (VI) increased the iodine content in leaves, but Se (IV) decreased. Furthermore, I (-I) had no effect on the uptake of Se in sprouts and roots, but I (V) reduced its uptake. In addition, both I and Se in the soaking solution affected the activity of the electron transport system, tocopherol content, and glutathione content [170]. Nevertheless, the interaction of iodine and selenium in sprouts remains to be investigated. Furthermore, a recent study found that the combination of sucrose (10 mg/L) and selenium (1.25 mg/L) treatment could more effectively increase vitamin C, sucrose, and fructose levels, especially the selenium level, compared with selenium treatment alone [172]. In addition, the combined application of sucrose (10 mg/L) and selenium (1.25 mg/L) more effectively regulated glucose metabolism and promoted the nutritional quality than selenium treatment alone [172]. Moreover, the enrichment of zinc in seeds should be performed with caution to avoid potential health risks of sprouts, such as zinc toxicity. The highest safe concentration of ZnSO_4_ used for treating pea seeds was 10 μg/mL, at which concentration the germination rate of the pea seeds was as high as 85% and the germination index was 154% [171]. Nevertheless, the bioavailability and safety of these minerals in biofortified pea sprouts and microgreens still need to be studied in animals and humans.

### 5.3. Pea Flour-Incorporated Products

Pulse flours can be added to food to promote their functional properties and nutritional content as well as product quality [173]. Pea flours could be incorporated into the wheat bread formulation to promote the quality and nutritional properties of the final products [174]. It was found that 10% substitution of wheat flour with pea flour had no adverse effects on dough rheology, bread texture, staling kinetics, or the product’s sensory attributes, while the incorporation of pea flours could enhance the nutritional quality of the final product by enhancing the contents of proteins and dietary fiber, and reducing the starch digestibility rates. In addition, it was found that the incorporation of 5% yellow pea flour into whole wheat flour could lead to a similar bread quality as that with only whole wheat flour [173]. Indeed, the composite flour containing yellow pea flour had overall better potential for bread making by providing good dough handling properties and good product quality, and promoting the nutritional value of the whole wheat bread. Furthermore, the effect of substituting 30% of the wheat flour with pea flour (raw materials, germinated peas, and toasted peas) on the dough properties and baking characteristics of white bread were evaluated [175]. It was found that the content of proteins obviously improved after the substitution of pea flour at 30%, and the bread formulated with toasted pea flour could result in similar properties to wheat flour bread. In addition, this study clearly demonstrated that high-quality bread could be achieved at a flour substitution level of up to 30% of pea flour, which provided the consumer with alternative bread with enhanced protein content and other nutritive properties. Moreover, pea flour has also been incorporated into wheat flour in the formulation of baked crackers [176]. Yellow and green pea flours were mixed with wheat flour at the level of up to 40% to make chemically leavened crackers [176]. It was found that the substitution of wheat flour with 40% pea flour notably improved the protein content and total dietary fiber content. Furthermore, the phenolic content and antioxidant capacity were also enhanced with the addition of pea flours. In addition, it was found that consumers preferred yellow pea cookies to green pea cookies. These results suggest that pea flour substitution has the potential to improve the nutrients and eating quality of wheat-based products.

Furthermore, purified pea fractions, such as pea protein isolates and starches, could be applied for the preparation of sponge cakes. A study was carried out for the investigation of the in vitro digestion properties of purified pea fractions (protein isolates and starches) in sponge cakes compared to unrefined pea flours and a wheat-based reference [177]. It was found that the complete substitution of wheat flour with either pea flours or combined pea proteins and pea starches resulted in pea-based cakes with more nutritional characteristics than those of wheat cakes. More specifically, the digestive rate of proteins in the wheat cake was more rapidly than that of proteins in pea-based cake. Additionally, cakes prepared from wheat flours or maize starches were more susceptible to α-amylase compared to those made from purified pea starches or whole pea flours, which may be owing to the high ratio of amylose and resistant starches in the pea cakes. Interestingly, similar digestion behaviors were observed in cakes made from the purified pea components and unrefined whole pea flours. Collectively, these results reveal that pea ingredients and whole pea flours have good potential for application in plant-based alternatives to wheat flours for utilization in gluten-free baked products.

Moreover, pea pods are usually regarded as by-products deriving from the pea processing industry, which are potential sources of dietary fiber, proteins, polyphenols, and minerals. A recent study utilized pea pod powder for the development of instant pea soup powders [178]. It was found that the instant pea soup powder containing 12.5% of pea pod powder was the most acceptable, with an overall acceptability of 8.5. In addition, the produced instant pea soup powder had high contents of dietary fiber (13.25%), carotenoids (6.65 mg/100 g), and chlorophyll (1.95 mg/100 g). Therefore, more studies remain to be carried out to leverage pea by-products for their valorisation in the food industry.

### 5.4. Meat Alternatives

Pea components, such as proteins, starches, and fiber, have been utilized in the development of various forms of meat and meat alternatives [29,179,180]. Pea proteins may detract from food properties but have shown excellent properties in the development of manufactured meat products [29]. For example, the addition of pea protein isolates (PPIs) in fortified beef patties could reduce their hardness, stickiness, gumminess, and chewiness, providing them with a softer texture that can meet the dietary target requirements for proteins in a population of older people [181]. The addition of PPIs in chicken nugget formulations could increase the nutritional value while reducing the high cost of the meat product [182]. The addition of PPIs in meat formulations not only promotes the nutritional value but also provides a vehicle to enhance the application of PPIs to maintain target protein intake. Furthermore, pea protein isolates could be applied for the production of fibrous meat alternatives by high-moisture extrusion cooking treatment [183].

Fiber plays a critical role in the texture of meat alternatives. Pea fiber has been added as a partial substitute for meat (to reduce the cost of the product) or fat (to develop a health product) in beef burgers [179]. It was found that there were no significant differences in pH, color parameters, texture profile, cooking loss, size reduction, or sensory acceptance among different beef burgers with pea fiber [179]. In addition, the impact of pea fiber on the quality properties of chicken meatballs was studied [184]. It was found that the addition of pea fiber in the chicken meatballs could increase their pH, yield, and moisture retention, while decreasing the diameter reduction and fat absorption. In particular, the addition of 3%, 6%, and 9% of pea fiber was found to be more successful in the production of chicken poultry. In addition, the addition of pea fiber in meatballs could obtain a more crumbly, firm, and gritty texture with increasing doses of fiber [185]. Indeed, subjective appetite sensations were not affected by the addition of pea fiber, and the addition of pea fiber could promote the nutritional composition of meatballs. Furthermore, pea starches and pea fiber have been applied for the substitution of wheat crumb in beef burgers [180]. It was found that the incorporation of a suitable ratio of pea starches and fiber into beef burgers could result in optimal firmness and juiciness characteristics, but without any adverse effects on consumer acceptability. Collectively, pea fiber is promising as a partial substitute for meat and fat due to the preservation of technical parameters and organoleptic acceptability.

### 5.5. Encapsulation and Packing Materials

Pea proteins can be used as an effective encapsulation system. The application of pea proteins in the emulsification and encapsulation of food bioactive ingredients has been recently summarized [186]. Recent investigations have studied the application of pea proteins as the single carrier or in combination with other biopolymers for the encapsulation of polyphenols, such as curcumin, quercetin, resveratrol, and hesperidin [187,188,189,190]. For instance, pea protein isolate (PPI) was successfully used as a nanocarrier for encapsulation of lipophilic polyphenols, such as curcumin, quercetin, and resveratrol [190]. It was found that curcumin, quercetin, and resveratrol were encapsulated in the PPI mainly driven by hydrophobic interaction or hydrogen bonding effects. After loading into the PPI, the environmental stability of three polyphenols towards UV light and thermal processing was enhanced, and the antioxidant potential of three polyphenols was also improved. In addition, PPI could be combined with high methoxylated pectin (HMP) to form electrostatic nanocomplexes for the encapsulation of quercetin and hesperetin [187,188]. The optimal conditions required for electrostatic nanocomplex formation were a 1:1 ratio of PPI to HMP and a pH of 4, which greatly increased the solubility, bioaccessibility, and antioxidant activity of hesperetin [187]. Another study investigated the encapsulation efficiency, antioxidant properties, stability, and release of quercetin (Que) encapsulated in soluble PPI-HMP nanocomplexes fabricated at pH 5 and pH 6 [188]. It was found that PPI-HMP nanocomplexes fabricated at pH 5 had higher encapsulation efficiency and loading capacity, while the nanocomplexes fabricated at pH 6 had higher antioxidant capacity. In addition, PPI-HMP-Que fabricated at pH 6 exhibited a greater protecting effect and higher release percentage of Que in the simulated gastrointestinal fluid [188]. Collectively, these results can provide useful information for fabricating PPI-based nanocarriers for the effective encapsulation of bioactive ingredients in the food industry.

Different vegetable proteins and animal proteins are regarded as excellent materials for the preparation of edible or non-edible coatings and films [191]. Pea proteins are widely utilized in the production of edible films and coating materials. Pea-protein-based films could provide high oxygen barrier properties but have high water vapor permeability due to their hydrophilic nature. The addition of corn starch nanocrystals (SN) and high-pressure homogenization (HPH) treatment can greatly promote the packaging properties of pea-protein-based films [192,193]. It was found that the addition of SN up to 10% could reduce the water vapor permeability and mechanical properties, and improve the thermal stability of pea-protein-based films [193]. In addition, HPH treatment could reduce the particle size and surface charge of PPI, and increase the surface hydrophobicity and free sulfhydryl of PPI, thereby supplying great potential for covalent bonding during film formation [192]. On the other hand, HPH treatment could decrease the opacity of PPI-based films from 7.39 to 4.82 at a pressure of 240 MPa, with a more homogeneous surface. After the HPH treatment, the tensile strength and elongation at break of the PPI-based films increased from 0.76 MPa to 1.33 MPa, and from 96% to 197%, respectively [192].

Furthermore, cellulose nanocrystals (CNC) isolated from pea hulls can enhance some packaging properties of carboxymethyl cellulose (CMC)-based films and chitosan (CS)-based films [194,195]. It was found that the addition of 5% CNC in the CMC-based films could improve the UV and water vapor barriers, mechanical strength, and thermal stability. Compared with CMC-based films, the CNC-CMC composite films showed an increase of 50.8% in tensile strength and a decrease of 53.4% in water vapor permeability. On the other hand, the CNC-reinforced CMC films could reduce the weight loss and maintain the content of vitamin C of red chilies [194]. In addition, the CNC derived from pea hulls could also promote the packaging properties of CS-based films [195]. The tensile strength of CS-based films was increased by 41% with the addition of 10% of CNC. In addition, the UV and water barrier properties of the CS-CNC composite films were improved. Moreover, in addition to pea proteins and fiber, pea starches can also be used for the development of packaging materials. Pea starches and polylactic acids are promising alternatives to petroleum-based plastics [196,197]. Recently, biodegradable films derived from pea starches and polylactic acids with a double-layer structure have been developed, which showed better toughness, thermal stability, and barrier capacity than those of polylactic acid-based films. On the other hand, the films could reduce the weight loss ratio of cherry tomatoes and extend the retention of organic acids and vitamin C. Collectively, these results suggest that pea proteins, fiber, and starches possess good potential to be used as encapsulation and packing materials in the food system.

## 6. Conclusions and Perspectives

Whole peas are rich in macronutrients, including proteins, starches, dietary fiber, and non-starch polysaccharides. Polyphenols, especially flavonoids and phenolic acids, are important bioactive ingredients that are mainly distributed in the pea coats. Anti-nutritional factors, such as phytic acid, lectin, and trypsin inhibitors may hinder nutrient absorption. Whole pea seeds can be processed by different techniques such as drying, milling, soaking, and cooking to improve their functional properties. In addition, the physicochemical and functional properties of pea starches and pea proteins can be improved by chemical, physical, enzymatic, and their combined modification methods. Owing to the multiple bioactive ingredients in peas, the pea and its products exhibit various health benefits, such as antioxidant, anti-inflammatory, antimicrobial, anti-renal fibrosis, and regulation of metabolic syndrome effects. Peas have been processed into various products such as pea beverages, germinated pea products, pea flour-incorporated products, pea-based meat alternatives, and encapsulation and packing materials. Collectively, the pea can be a functional food that complements other grains for various applications in the food system.

Recent studies identified the detailed chemical structures of several bioactive polyphenols and peptides, while the structural information regarding the soluble dietary fiber is limited. Although various methods have been carried out for the processing of whole pea seeds, studies on the effects of different processing techniques on the nutritional quality and bioactive components of pea seeds are still limited. Many studies have revealed that pea and its bioactive components exhibit various health-enhancing benefits, while most studies only used crude extracts. It is important to establish efficient methods to isolate and purify specific bioactive compounds from pea and its by-products. In addition, several studies have investigated the possible mechanisms of action of pea and its bioactive components, while related molecular targets still need to be revealed. The bioactivities of individual polyphenols and dietary fiber from peas are still unclear, requiring more study in the future. Fortification of pea sprouts and microgreens with essential elements is one of the effective ways to promote their nutritional value. Nevertheless, the bioavailability and safety of these minerals in biofortified pea sprouts remain to be clarified. The potential of pea proteins and pea fiber in the development of meat alternatives should also be further explored to meet the increasing demand. Finally, the pea pods or hulls, an under-utilized agricultural by-product, are also a source of polyphenols and dietary fiber, but there is less information on their use in functional foods. Possible production methods should be developed to add value to these by-products.

## Figures and Tables

**Table 2:** Biological activities of pea and its components, and their potential mechanisms of action.

Sample Types	Experimental Models	Major Results	References
**Antioxidant effect**			
Seed flour extracted with 95% ethanol	In vitro (DPPH)	➢ The IC_50_ values for DPPH free radical scavenging activity were varied in different microwave drying, ranging from 0.1 to 0.9 mg/mL ➢ Microwave drying at 100 W had the highest DPPH free radical scavenging activity among all drying conditions	[74]
Seed flour extracted with 80% ethanol	In vitro (ABTS; DPPH; reducing power) In vitro (cell model, OA-induced HepG2 cells)	➢ The DPPH free radical scavenging efficiency, ABTS free radical scavenging efficiency, and reducing power of pea extracts were 56.58%, 27.25%, and 0.222 at a concentration of 1.0 mg/mL, respectively ➢ ↑ SOD, GSH-Px; ↓ MDA, ROS	[65]
Seed flour extracted with 80% methanol	In vitro (ABTS; DPPH)	➢ A positive correlation was observed between the TPC and ABTS free radical scavenging activity (r = 0.59) ➢ While an inverse trend was observed for DPPH free radical scavenging activity (r = −0.31)	[82]
Seed flour extracted with mixed solution (acetone/water/acetic acid, 70:29.5:0.5, *v*/*v*/*v*)	In vitro (ABTS; FRAP)	➢ The ABTS free radical scavenging capacity of 75 pea varieties ranged from 3.04 to 22.27 μM TE/g ➢ The FRAP of 75 pea varieties ranged from 1.24 to 18.87 mM Fe^2+^/g DW ➢ Dark pea seeds (e.g., brown, purple, dark and brown with strap or dotted) contained more TPC, TFC, ABTS, and FRAP than light-colored seeds (e.g., cream, green).	[68]
Seed coat extracted with mixed solution (methanol/water/acetic acid mixture, 80:19:1, *v*/*v*/*v*)	In vitro (DPPH; FRC; FCC)	➢ The DPPH free radical scavenging activity ranged from 0.72 to 2.55 mM TE/g, exhibiting a good correlation with TPC values (r = 0.971) ➢ The FRC absorbance values at 700 nm ranged from 0.019 to 0.312 ➢ The FCC ranged from 29.6 to 75.8%	[125]
Seed coat extracted with mixed solution (acetone/water/acetic acid mixture, 80:19:1, *v*/*v*/*v*)	In vitro (DPPH; FRC; FCC)	➢ The DPPH free radical scavenging activity ranged from 0.54 to 8.04 mM TE/g ➢ The FRC ranged from 0.021 to 0.346 ➢ The FCC ranged from 8.1% to 33.7%	[126]
Seed coat extracted with water, methanol, and ethyl acetate	In vitro (ABTS; DPPH; FRAP)	➢ The IC_50_ values of ABTS free radical scavenging activity of ethyl acetate extract (9.61 μM TEAC/g) was higher than that of the methanol extract (1.9 μM TEAC/g) ➢ The IC_50_ values of DPPH free radical scavenging activity were 350 μg/mL and 650 μg/mL for ethyl acetate and methanol extract, respectively ➢ The FRAP of methanol, water, and ethyl acetate extracts, at a concentration of 2 mg/mL, were 1.32, 0.36, and 1.84, respectively	[127]
Red and yellow pea hull in vitro digestion products	In vitro (DPPH; ABTS; H_2_O_2_; FRAP)	➢ Strong correlations existed in red hulls between their antioxidant activities (DPPH; ABTS; H_2_O_2_; FRAP) and TPC/TFC (r > 0.92)	[69]
Pea sprout extracted with 80% methanol	In vitro (DPPH; ORAC; CUPRAC)	➢ The 7th day of germination showed the strongest DPPH free radical scavenging activity (512.64 mg TE/100 g DW), ORAC value (6083.54 mg TE/100 g DW), and CUPRAC inhibition (44.05%) among all tested samples	[70]
Pea hull extracted with 95% ethanol	In vitro (DPPH; reducing power; FRAP)	➢ The DPPH free radical scavenging activity ranged from 76.55% to 91.03% ➢ The reducing power ranged from 0.5 to 0.63 ➢ The FRAP values ranged from 0.25 to 0.34 mmol/L	[128]
Peptides derived from pea protein hydrolysate	In vitro (DPPH; OH)	➢ The second fraction (F1-2) purified from hydrolysates (<1 kDa) had the highest DPPH radical scavenging rate of 37.94% and OH free radical scavenging rate of 28.43% among all samples	[129]
Whole seed flour	In vivo (HFD-induced Sprague–Dawley (SD) male rats)	➢ ↑ GSH-Px, SOD, and T-AOC ➢ ↑ Nrf2, NQO1, CAT, HO-1 ➢ ↓ MDA	[65]
Seed coat extracted with water	In vivo (DOX-induced albino male rats)	➢ ↑ SOD, GPX, CAT; ↑ GSH ➢ ↓ MDA, NO	[72]
Green pea hull extracted with 80% methanol	In vivo (D-galactose-induced SD female rats)	➢ ↑ T-AOC, SOD, GSH-Px; ↑ GSH ➢ ↓ MDA	[130]
Yellow pea hull extracted with 80% methanol	In vivo (D-galactose-induced SD female rats)	➢ ↑ T-AOC, SOD, GSH-Px; ↑ GSH ➢ ↓ MDA	[73]
**Anti-inflammatory effect**			
Green pea hull in vitro digestion products	In vitro (LPS-induced Caco-2/Raw264.7 cells coculture)	➢ ↓ NO, IL-6, TNF-α ➢ ↓ COX-2, iNOS	[131]
Peptides derived from pea protein hydrolysate	In vitro (LPS/IFN-γ-induced RAW 264.7 cells)	➢ ↓ NO, IL-6, TNF-α	[132]
Whole seed flour	In vivo (DSS-induced colitis in HFD-fed C57BL/6J female mice)	➢ ↓ IL-6, IL-17, IFN-γ ➢ ↓ iNOS, COX-2, MCP-1 ➢ ↑ MUC-2, goblet cell differentiation markers (e.g., Tff3, Klf4, Spdef1)	[133]
Green pea hull extracted with 80% ethanol	In vivo (DSS-induced colitis in C57BL/6 male mice)	➢ ↓ NO, IL-6, TNF-α ➢ ↓ COX-2, iNOS ➢ ↓ Keap1; ↑ Nrf2 ➢ ↑ SCFAs (e.g., acetic acid, propionic acid, and *n*-butyric acid) ➢ ↑ *Lactobacillaceae*, *Lachnospiraceae*, *Firmicutes*; ↓ *Bacteroidetes*	[77]
Two pea seed albumin extracts (PSE/AF-PSE)	In vivo (DSS-induced colitis in C57BL/6J male mice)	➢ PSE: ↓ IFN-γ, IL-6, IL-12, TNF-α; ↓ iNOS, COX-2, MMP-2, MMP-9, MMP-14, ICAM-1; ↑ MUC-3, ZO-1, occluding; ↓ TLR-2, -4, -6, -9 ➢ AF-PSE: ↓ IL-6, IL-12; ↓ COX-2, MMP-9 and -14, ICAM-1; ↓ TLR-4, -6, -9	[134]
**Regulation of metabolic syndrome**			
***Anti-hypertensive activity***			
Peptides derived from pea protein hydrolysate	In vitro (ACE inhibition assay)	➢ The peptide fraction (B) had the highest ACE inhibitory activity (IC_50_ = 0.073 mg/mL) among all tested samples	[135]
Peptides derived from pea protein hydrolysate	In vitro (A7r5 cells)	➢ A peptide with the sequence of AKSLSDRFSY was identified as a novel ACE2 upregulating peptide	[136]
Tripeptide (Leu-Arg-Trp)	In vitro (A7r5 cells)	➢ ↑ ACE2 and MasR to upregulate the ACE2-Ang-(1-7)-MasR axis	[137]
Peptides derived from pea protein hydrolysate	In vitro (ACE and renin inhibition assays) In vivo (male SHRs)	➢ The peptide fraction 7 had the highest dual inhibitory effects of renin and ACE with 52.16% and 95.17% inhibition rates, respectively ➢ The renin and ACE inhibitory activities of synthesized peptides were ranged from 17.4 to 49.9% and 5.72 to 87.54%, respectively, and three of them (LTFPG, IFENLQN, and FEGTVFENG) showed strong ACE and renin inhibitory activities ➢ LTFPG acted the fastest in reducing SBP of SHR with a maximum of −37 mmHg after 2 h of oral administration	[28]
Peptides derived from pea protein hydrolysate	In vitro (ACE and renin inhibition assays) In vivo (male SHRs)	➢ The IC_50_ values of inhibition on renin and ACE were 0.57 and 0.10 mg/mL, respectively ➢ The maximum decrease in SBP was −36 mmHg after 2 or 4 h, becoming −18 mmHg after 24 h ➢ The maximum decrease in SBP after 3 weeks was −22 or −26 mmHg with partial substitution of casein 0.5% or 1% (*w*/*w*) in the SHR diet, respectively	[138]
***Hypolipidemic activity***			
Pea pod autoclaved extract (AE)	In vitro (pancreatic lipase inhibition and cholesterol adsorption capacity assay) In vivo (high-sucrose-induced SD male rats)	➢ AE at 13.3 mg/mL significantly inhibited the pancreatic lipase activity by more than 40% compared to 0 mg/mL ➢ 2000 mg of AE adsorbed approximately 33% of cholesterol ➢ ↑ Total lipid, TG and TC in feces ➢ ↓ Serum TC, TG ➢ ↑ Bifidobacterial; ↓ Clostridia	[139]
Pea seed flour	In vivo (HFD-induced male SD rats)	➢ ↓ Serum TC, LDL-C; ↑ HDL-C	[65]
Pea protein isolate	In vivo (HFD-induced male SD rats)	➢ ↓ Plasma TC, TG ➢ ↑ LDL receptor ➢ ↓ SREBP-1c and the target genes (FAS, SCD1, SCD2)	[140]
***Anti-obesity activity***			
Pea protein hydrolysate	In vitro (3T3-L1 preadipocytes subline)	➢ ↑ Lipid accumulation; ↑ glucose uptake ➢ ↑ GLUT4, adiponectin, aP2, PPARγ; ↓ Pref-1 ➢ ↑ PPARγ ligand activity	[141]
Pea flour and dietary fiber	In vivo (HFHSD-induced obese SD male rats)	➢ ↓ Weight gain, final percent body fat, fasting blood glucose; ↑ oral glucose tolerance ➢ ↓ *Firmicutes*/*Bacteroidetes* ratio ➢ ↓ *Clostridium leptum*	[55]
Pea fiber	Clinical trial (12-week single center, double-blind placebo-controlled trial with 53 adults with overweight or obesity)	➢ Fecal SCFAs (↑ acetate; ↓ isovalerate) ➢ Fecal BAs (↓ cholic acid, chenodeoxycholic acid, deoxycholic acid) ➢ ↑ *Lachnospira*; ↓ *Actinomyces*, *Holdermania*, *Oscillospira* ➢ The change in body weight of participants showed a negative correlation with their change in *Lachnospira* (r = −0.463) abundance	[51]
***Anti-diabetic effect***			
Pea protein hydrolysate	In vitro (α-amylase and α-glucosidase inhibition assays)	➢ The highest inhibitory activity against α-amylase among all samples was 30.52% at the concentration of 225 μg/mL ➢ The highest inhibitory activity against α-glucosidase among all samples was 53.35% at the concentration of 20 mg/mL	[142]
Purified pea glycoproteins (PGP1, PGP2, and PGP3)	In vitro (α-amylase and α-glucosidase inhibition assays)	➢ The inhibition of α-amylase with PGP1 was only about 2.73% at a concentration of 2 mg/mL, while the inhibition rates of PGP2 and PGP3 were 8.71% and 22.34%, respectively ➢ The inhibition of α-glucosidase was most potent with the product of gastric digestion, which showed an increase of about 45.87% compared to undigested PGP2 at the same concentration	[143]
Purified pea glycoprotein (PGP2)	In vivo (STZ-induced diabetic ICR male mice)	➢ ↓ Weight loss, food intake, fasting blood glucose; ↑ oral glucose tolerance, insulin secretion ➢ ↓ Serum TC, TG, LDL-C; ↑ HDL-C; ↓ glycated serum protein ➢ ↓ Insulin resistance index; ↑ β-cell function index, insulin sensitivity index ➢ ↑ IRS-1, IRS-2, GLUT1	[144]
Pea oligopeptide	In vivo (HFD and STZ-induced diabetic Kunming male mice)	➢ ↓ Weight loss, fasting blood glucose; ↑ oral glucose tolerance ➢ ↑ Serum insulin level; ↓ serum TC, TG, FAA; ↑ HDL-C ➢ ↓ Liver fat deposition ➢ ↑ The muscle and liver glycogen ➢ Protect the liver and kidney structures	[145]
Pea dietary fiber	In vivo (STZ-induced diabetic Balb/c male mice)	➢ ↓ Blood glucose, weight loss; ↑ oral glucose tolerance ➢ ↓ TC, TG ➢ The pancreatic islet morphology was improved	[50]
Pea protein	Clinical trial (a randomised controlled trial with a high-carbohydrate beverage intake in healthy individuals)	➢ ↓ Glucose incremental area under the curve (iAUC180)	[146]
**Antimicrobial effect**			
11S pea globulin (11SGP)	In vitro Bacteria: *Bacillus cereus*, *Listeria monocytogenes*, *Streptococcus pyogenes*, *Escherichia coli*, *Acinetobacter baumannii*, and *Pseudomonas aeruginosa*; Fungi: *Alternaria alternate*, *Aspergillus flavus*, *Fusarium oxysporum*, and *Monascus purpureus*	➢ The minimum inhibitory concentrations of 11SGP against bacteria ranged from 120 to 190 μg/mL ➢ The minimum inhibitory concentrations of 11SGP against fungi ranged from 55 to 80 μg/mL	[147]
Pea lectin	In vitro Bacteria: *Staphylococcus aureus*, *Streptococcus mutants*, *Pseudomonas aeruginosa*, and *Klebsiella pneumonia* Fungi: *Candida albicans*	➢ The minimum inhibitory concentrations of pea lectin (90% fractions) against bacteria ranged from 62.5 to 125 μg/mL ➢ The minimum inhibitory concentration of pea lectin (90% fractions) against *C. albicans* was 250 μg/mL	[148]
Pea peel extracted with water, methanol, and ethyl acetate	In vitro Bacteria: *Staphylococcus aureus,* *Salmonella enterica*, *Escherichia coli*, and *Pseudomonas aeruginosa* Fungi: *Aspergillus niger* and *Candida albicans*	➢ The ethyl acetate extract of pea peel exhibited the strongest antimicrobial activity among all tested samples ➢ The minimum inhibitory concentrations of ethyl acetate extract against bacteria ranged from 350 to 850 μg/mL ➢ The minimum inhibitory concentrations of ethyl acetate extract against fungi ranged from 450 to 550 μg/mL	[127]
Pea pod polysaccharide	In vitro Bacteria: *Bacillus thuringiensis*, *B. subtilis*, *Actinomycete* sp., *Enterococcus faecalis*, *Listeria monocytogenes*, *Micrococcus luteus*, *Klebsiella pneumonia*, *Pseudomonas aeruginosa*, and *Salmonella Typhimirium*	➢ The largest zones of inhibition were detected against *B. subtilis, B. thuringiensis* and *M. luteus* with inhibition zones of 16, 15, and 15 mm at the concentration of 50 mg/mL, respectively ➢ The greatest inhibition was detected against *P. aeruginosa* with an inhibition zone of 15 mm at the concentration of 50 mg/mL	[149]
**Anti-renal fibrosis effect**			
Peptides derived from pea protein hydrolysate	In vitro (glucose-induced MES13 SV40 cells)	➢ ↓ TGF-β1, SMAD2, SMAD3, SMAD4; ↑ SMAD7	[150]
Peptides derived from pea protein hydrolysate	In vitro (glucose-induced MES13 SV40 cells)	➢ ↑ Cell proliferation; ↓ FN, TGF-β1	[151]
**Anti-cancer effect**			
Pea seed coat extracted with water	In vitro (cell lines, human colon denocarcinoma LS174, breast carcinoma MDA-MB-453, lung carcinoma A594, and myelogenous leukemia K562)	➢ MDA-MB-453, IC_50_ values ranged from 0.89% up to above 10.0% ➢ LS174, IC_50_ values ranged from 1.84% up to above 10.0% ➢ A549, IC_50_ values ranged from 1.17% up to above 10.0% ➢ K562, IC_50_ values ranged from 0.41% up to above 10.0%	[126]
Pea lectin	In vitro (cell line, Ehrlich ascites carcinoma (EAC) cells) In vivo (Ehrlich ascites carcinoma cells in adult Swiss albino mice)	➢ Pea lectin showed 11.7–84% inhibitory effect against EAC cells at the concentration range of 8–120 μg/mL as determined by MTT assay ➢ Pea lectin showed 63% and 44% growth inhibition against EAC cells in vivo when administered 2.8 mg/kg/day and 1.4 mg/kg/day ➢ Pea lectin can arrest the cell cycle at G2/M phase ➢ ↑ *Bax* gene expression; ↓ *Bcl-2* and *Bcl-X* gene expression	[152]
**Immunomodulatory effect**			
Peptides derived from pea protein hydrolysate	In vivo (BALB/c female mice)	➢ ↑ Phagocytic activity ➢ ↑ IgA+, IL-4+, IL-10+, IFN-γ+ cells ➢ ↑ IL-6 via ↑ TLR2 and TLR4	[132]
**Anti-osteoporosis effect**			
Pea tripeptide (Leu-Arg-Trp)	In vitro (MC3T3-E1 cell)	➢ ↑ Cell proliferation, osteoblastic differentiation, matrix mineralization, ➢ ↑ Runx2, ALP, COL1A2, phosphorylation Akt ➢ ↓ Osteoclastogenesis	[153]
**Anti-fatigue effect**			
Peptides derived from pea protein hydrolysate	In vivo (Kunming mice)	➢ ↑ Swimming times ➢ ↑ Muscle glycogen, hepatic glycogen, insulin level, lactate dehydrogenase activity ➢ ↓ Blood urea nitrogen, blood lactic acid ➢ ↑ SOD, GSH-Px; ↓ MDA ➢ ↑ Phagocytic activity, sIgA secretion; ↓ IL-6, TNF-α	[154]

ABTS, 2,2′-azino-bis (3-ethylbenzothiazoline-6-sulphonic acid); ACE, angiotensin I-converting enzyme; AF-PSE, albumin fraction from pea seed extract; ALP, alkaline phosphatase; ALT, alanine amino transferase; Ang II, angiotensin II; aP2, adipocyte fatty acid-binding protein; AST, aspartate amino transferase; BAs, bile acids; Bcl, B-cell lymphoma; CAT, catalase; COL1A2, alpha-2 type I collagen; COX-2, cyclooxygenase-2; CUPRAC, cupric reducing antioxidant capacity; DOX, doxorubicin hydrochloride; DPPH, 2,2-Diphenyl-1-picrylhydrazyl; DSS, dextrane sodium sulphate; EAC, Ehrlich ascites carcinoma; FAA, free amino acid; FAS, fatty acid synthase; FCC, ferrous ion-chelating capacity; FN, fibronectin; FRAP, ferric reducing antioxidant power; FRC, ferric ion-reducing capacity; GLUT, glucose transporter; GSH, glutathione; GSH-Px, glutathione peroxidase; HDL, high-density lipoprotein; HDL-C, high-density lipoprotein cholesterol; HFD, high-fat diet; HFHSD, high-fat/high-sucrose diet; H_2_O_2_, hydrogen peroxide; HO-1, heme oxygenase 1; ICAM, intercellular adhesion molecule; IFN-γ, interferon-gamma; IgA+, immunoglobulin class A+; IgG, immunoglobulin class G; IL, interleukin; iNOS, inducible nitric oxide synthase; IRS, insulin receptor substrate; Keap1, Kelch-like ECH-associated protein 1; Klf4, Kruppel-like factor 4; LDL, low-density lipoprotein; LDL-C, low-density lipoprotein cholesterol; LPS, lipopolysaccharide; MCP, monocyte chelator protein; MDA, malondialdehyde; MMP, metalloproteinase; NO, nitric oxide; NQO1, NAD(P)H quinone dehydrogenase 1; Nrf2, transcription factor NF-E2-related factor 2; OA, oleic acid; OH, hydroxyl; ORAC, oxygen radical absorbance capacity; PPARγ, peroxisome proliferator-activated receptor γ; Pref, preadipocyte factor; PSE, pea seed extract; ROS, reactive oxygen species; Runx2, runt-related transcription factor; SBP, systolic blood pressure; SCD, stearoyl-CoA desaturase; SCFAs, short-chain fatty acids; SHR, spontaneously hypertensive rat; SOD, superoxide dismutase; Spdef1, SAM pointed domain ETS factor 1; SREBP, sterol regulatory element-binding protein; STZ, streptozotocin; T-AOC, total antioxidant capacity; TC, total cholesterol; TFC, total flavonoid content; Tff3, trefoil factor 3; TG, triglyceride; TGF-β1, transforming growth factor beta; TLR, toll-like receptors; TNF-α, tumor necrosis factor α; TPC, total phenolic content.

## Data Availability

Data are contained within the article.

## Abbreviations

A594, lung carcinoma; ABTS, 2,2 azinobis 3-ethylbenzo-thiozoline-6-sulfonic acid; ACE, angiotensin I-converting enzyme; ADIPOR, adiponectin receptor; AF-PSE, albumin fraction from pea seed extract; Akt, protein kinase B; ALP, alkaline phosphatase; ALT, alanine amino transferase; AMPK, adenosine 5′-monophosphate (AMP)-activated protein kinase; Ang, angiotensin; aP2, adipocyte fatty acid-binding protein; AST, aspartate amino transferase; BAs, bile acids; Bcl, B-cell lymphoma; CAT, catalase; COL1A2, alpha-2 type I collagen; COX-2, cyclooxygenase-2; CUPRAC, cupric reducing antioxidant capacity; DOX, doxorubicin hydrochloride; DPPH, 2,2-Diphenyl-1-picrylhydrazyl; DSS, dextran sodium sulphate; EAC, Ehrlich ascites carcinoma; ESI, electrospray ionization; FAA, free amino acid; FAS, fatty acid synthase; FBG, fasting blood glucose; FCC, ferrous ion-chelating capacity; FN, fibronectin; FRAP, ferric reducing antioxidant power; FRC, ferric ion-reducing capacity; GLUT, glucose transporter; GSH, glutathione; GSH-Px, glutathione peroxidase; HDL, high-density lipoprotein; HDL-C, high-density lipoprotein cholesterol; HFD, high-fat diet; HFHSD, high-fat/high-sucrose diet; H2O2, hydrogen peroxide; HO-1, heme oxygenase 1; HRMS, high-resolution mass spectrometry; ICAM, intercellular adhesion molecule; IFN-γ, interferon-gamma; IgA+, immunoglobulin class A+; IgG, immunoglobulin class G; IKBA, inhibitory kBa; IL, interleukin; iNOS, inducible nitric oxide synthase; IRS, insulin receptor substrate; K562, myelogenous leukemia; Keap1, Kelch-like ECH-associated protein 1; Klf4, Kruppel-like factor 4; LC, liquid chromatography; LDL, low-density lipoprotein; LDL-C, low-density lipoprotein cholesterol; LDLR, low-density lipoprotein receptor; LPS, lipopolysaccharide; LS174, human colon denocarcinoma; LTQ, linear ion-trap quadrupole; MASR, Mas receptor; MCP, monocyte chelator protein; MDA, malondialdehyde; MDA-MB-453, breast carcinoma; MMP, metalloproteinase; MS, mass spectrometry; MS/MS, tandem mass spectrometry; NO, nitric oxide; NQO1, NAD(P)H quinone dehydrogenase 1; Nrf2, transcription factor NF-E2-related factor 2; OA, oleic acid; OGT, oral glucose tolerance; ·OH, hydroxyl radical; ORAC, oxygen radical absorbance capacity; P13K, phosphatidylinositol 3-kinase; PPARγ, peroxisome proliferator-activated receptor γ; Pref, preadipocyte factor; PSE, pea seed extract; Q, quadrupole; ROS, reactive oxygen species; Runx2, runt-related transcription factor; SBP, systolic blood pressure; SCD, stearoyl-CoA desaturase; SCFAs, short-chain fatty acids; SHR, spontaneously hypertensive rat; SOD, superoxide dismutase; Spdef1, SAM pointed domain ETS factor 1; SREBP, sterol regulatory element-binding protein; STZ, streptozotocin; T-AOC, total antioxidant capacity; TC, total cholesterol; TFC, total flavonoid content; Tff3, trefoil factor 3; TG, triglyceride; TGF-β1, transforming growth factor beta; TLR, toll-like receptors; TNF-α, tumor necrosis factor α; TPC, total phenolic content; UHPLC, ultrahigh-performance liquid chromatography.

## References

[1] Fahmi R., Ryland D., Sopiwnyk E., Aliani M. (2019). Sensory and Physical Characteristics of Pan Bread Fortified with Thermally Treated Split Yellow Pea (*Pisum sativum* L.) Flour. J. Food Sci..

[2] Han X., Akhov L., Ashe P., Lewis C., Deibert L., Irina Zaharia L., Forseille L., Xiang D., Datla R., Nosworthy M. (2023). Comprehensive Compositional Assessment of Bioactive Compounds in Diverse Pea Accessions. Food Res. Int..

[3] Kumari T., Deka S.C. (2021). Potential Health Benefits of Garden Pea Seeds and Pods: A Review. Legume Sci..

[4] Liu M., Wu N.-N., Yu G.-P., Zhai X.-T., Chen X., Zhang M., Tian X.-H., Liu Y.-X., Wang L.-P., Tan B. (2018). Physicochemical Properties, Structural Properties, and in vitro Digestibility of Pea Starch Treated with High Hydrostatic Pressure. Starch-Starke.

[5] Raghunathan R., Hoover R., Waduge R., Liu Q., Warkentin T.D. (2017). Impact of Molecular Structure on the Physicochemical Properties of Starches Isolated from Different Field Pea (*Pisum sativum* L.) Cultivars Grown in Saskatchewan, Canada. Food Chem..

[6] Gao L.C., Wu Y.X., Wan C.X., Wang P.K., Yang P., Gao X.L., Eeckhout M., Gao J.F. (2022). Structural and Physicochemical Properties of Pea Starch Affected by Germination Treatment. Food Hydrocolloid.

[7] Santos C.S., Carbas B., Castanho A., Vasconcelos M.W., Patto MC V., Domoney C., Brites C. (2019). Variation in Pea (*Pisum sativum* L.) Seed Quality Traits Defined by Physicochemical Functional Properties. Foods.

[8] Devi J., Sanwal S.K., Koley T.K., Mishra G.P., Karmakar P., Singha P.M., Singh B. (2019). Variations in the Total Phenolics and Antioxidant Activities among Garden Pea (*Pisum sativum* L.) Genotypes Differing for Maturity Duration, Seed and Flower Traits and their Association with the Yield. Sci. Hortic..

[9] Shi L., Arntfield S.D., Nickerson M. (2018). Changes in Levels of Phytic Acid, Lectins and Oxalates during Soaking and Cooking of Canadian Pulses. Food Res. Int..

[10] Wojdylo A., Nowicka P., Tkacz K., Turkiewicz I.P. (2020). Sprouts vs. Microgreens as Novel Functional Foods: Variation of Nutritional and Phytochemical Profiles and their in vitro Bioactive Properties. Molecules.

[11] Avezum L., Rondet E., Mestres C., Achir N., Madode Y., Gibert O., Lefevre C., Hemery Y., Verdeil J.-L., Rajjou L. (2022). Improving the Nutritional Quality of Pulses via Germination. Food Rev. Int..

[12] Babbitt C.W., Babbitt G.A., Oehman J.M. (2021). Behavioral Impacts on Residential Food Provisioning, Use, and Waste during the COVID-19 Pandemic. Sustain. Prod. Consum..

[13] Dahl W.J., Foster L.M., Tyler R.T. (2012). Review of the health benefits of peas (Pisum sativum L.). Br. J. Nutr..

[14] Yu Z., Fan Y.S., Wang X.W., Xia M., Cai Y. (2020). In Vitro and In Vivo Digestibility of Pea and Chickpea Powder Prepared by Cooking and Drying Treatment. Int. J. Food Prop..

[15] Jenkins D.J., Dehghan M., Mente A., Bangdiwala S.I., Rangarajan S., Srichaikul K., Mohan V., Avezum A., Díaz R., Rosengren A. (2021). Glycemic Index, Glycemic Load, and Cardiovascular Disease and Mortality. N. Engl. J. Med..

[16] Thakur S., Scanlon M.G., Tyler R.T., Milani A., Paliwal J. (2019). Pulse Flour Characteristics from a Wheat Flour Miller’s Perspective: A Comprehensive Review. Compr. Rev. Food Sci. Food Saf..

[17] Ge J., Sun C.X., Corke H., Gul K., Gan R.Y., Fang Y.P. (2020). The Health Benefits, Functional Properties, Modifications, and Applications of Pea (*Pisum sativum* L.) Protein: Current Status, Challenges, and Perspectives. Compr. Rev. Food Sci. Food Saf..

[18] Kan L., Nie S., Hu J., Wang S., Bai Z., Wang J., Zhou Y., Jiang J., Zeng Q., Song K. (2018). Comparative Study on the Chemical Composition, Anthocyanins, Tocopherols and Carotenoids of Selected Legumes. Food Chem..

[19] Ye S.X., Shah B.R., Li J., Liang H.S., Zhan F.C., Geng F., Li B. (2022). A Critical Review on Interplay Between Dietary Fibers and Gut Microbiota. Trends Food Sci. Technol..

[20] Ashokkumar K., Diapari M., Jha A.B., Tar’an B., Arganosa G., Warkentin T.D. (2015). Genetic Diversity of Nutritionally Important Carotenoids in 94 Pea and 121 Chickpea Accessions. J. Food Compos. Anal..

[21] Fahim J.R., Attia E.Z., Kamel M.S. (2019). The Phenolic Profile of Pea (*Pisum sativum*): A Phytochemical and Pharmacological Overview. Phytochem. Rev..

[22] Chen S.-K., Lin H.-F., Wang X., Yuan Y., Yin J.-Y., Song X.-X. (2023). Comprehensive Analysis in the Nutritional Composition, Phenolic Species and in vitro Antioxidant Activities of Different Pea Cultivars. Food Chem. X.

[23] Langyan S., Yadava P., Khan F.N., Dar Z.A., Singh R., Kumar A. (2022). Sustaining Protein Nutrition through Plant-Based Foods. Front. Nutr..

[24] Naghshi S., Sadeghi O., Willett W.C., Esmaillzadeh A. (2020). Dietary Intake of Total, Animal, and Plant Proteins and Risk of All Cause, Cardiovascular, and Cancer Mortality: Systematic Review and Dose-Response Meta-Analysis of Prospective Cohort Studies. BMJ.

[25] Ewy M.W., Patel A., Abdelmagid M.G., Elfadil O.M., Bonnes S.L., Salonen B.R., Hurt R.T., Mundi M.S. (2022). Plant-Based Diet: Is it as Good as an Animal-Based Diet When it Comes to Protein?. Curr. Nutr. Rep..

[26] Arif U., Ahmed M.J., Rabbani M.A., Arif A.A. (2020). Assessment of Genetic Diversity in Pea (*Pisum sativum* L.) Landraces Based on Physico-Chemical and Nutritive Quality Using Cluster and Principal Component Analysis. Pak. J. Bot..

[27] Abdel-Aal E.-S.M., Ragaee S., Rabalski I., Warkentin T., Vandenberg A. (2019). Nutrient Content and Viscosity of Saskatchewan-Grown Pulses in Relation to their Cooking Quality. Can. J. Plant. Sci..

[28] Aluko R.E., Girgih A.T., He R., Malomo S., Li H., Offengenden M., Wu J.P. (2015). Structural and Functional Characterization of Yellow Field Pea Seed (*Pisum sativum* L.) Protein-Derived Antihypertensive Peptides. Food Res. Int..

[29] Shanthakumar P., Klepacka J., Bains A., Chawla P., Dhull S.B., Najda A. (2022). The Current Situation of Pea Protein and its Application in the Food Industry. Molecules.

[30] Ge J., Sun C.X., Mata A., Corke H., Gan R.Y., Fang Y.P. (2021). Physicochemical and pH-Dependent Functional Properties of Proteins Isolated from Eight Traditional Chinese Beans. Food Hydrocolloid.

[31] Wang N., Hatcher D.W., Warkentin T.D., Toews R. (2010). Effect of Cultivar and Environment on Physicochemical and Cooking Characteristics of Field Pea (*Pisum sativum*). Food Chem..

[32] Chen X.Y., Ma X.W., Wen J.Y., Liu X.C., Yu X.R., Xiong F. (2021). Morphological, Structural and Functional Properties of Starches from Different Legume Resources. Legume Res..

[33] Ren Y.K., Setia R., Warkentin T.D., Ai Y.F. (2021). Functionality and Starch Digestibility of Wrinkled and Round Pea Flours of Two Different Particle Sizes. Food Chem..

[34] Ren Y., Yuan T.Z., Chigwedere C.M., Ai Y. (2021). A Current Review of Structure, Functional Properties, and Industrial Applications of Pulse Starches for Value-Added Utilization. Compr. Rev. Food Sci. Food Saf..

[35] Petropoulou K., Salt L., Warren F. (2017). A Seed Trait Studied by Gregor Mendel in *Pisum sativum* L. (Pea): Potential Prevention of Type 2 Diabetes. Legumes for Global Food Security.

[36] Chung H.J., Liu Q. (2012). Physicochemical Properties and In Vitro Digestibility of Flour and Starch from Pea (*Pisum sativum* L.) Cultivars. Int. J. Biol. Macromol..

[37] Wang M., Wichienchot S., He X., Fu X., Huang Q., Zhang B. (2019). In Vitro Colonic Fermentation of Dietary Fibers: Fermentation Rate, Short-Chain Fatty Acid Production and Changes in Microbiota. Trends Food Sci. Technol..

[38] Wang S.J., Sharp P., Copeland L. (2011). Structural and Functional Properties of Starches from Field Peas. Food Chem..

[39] Liu C., Wang S.J., Copeland L., Wang S. (2015). Physicochemical Properties and in vitro Digestibility of Starches from Field Peas Grown in China. LWT-Food Sci. Technol..

[40] Guo K., Lin L.S., Fan X.X., Zhang L., Wei C.X. (2018). Comparison of Structural and Functional Properties of Starches from Five Fruit Kernels. Food Chem..

[41] Ashogbon A.O., Akintayo E.T., Oladebeye A.O., Oluwafemi A.D., Akinsola A.F., Imanah O.E. (2021). Developments in the Isolation, Composition, and Physicochemical Properties of Legume Starches. Crit. Rev. Food Sci. Nutr..

[42] Wojeicchowski J.P., De Siqueira G.L.D.A., Lacerda L.G., Schnitzler E., Demiate I.M. (2018). Physicochemical, Structural and Thermal Properties of Oxidized, Acetylated and Dual-Modified Common Bean (*Phaseolus vulgaris* L.) Starch. Food Sci. Technol..

[43] Wani I.A., Sogi D.S., Gill B.S. (2015). Physico-Chemical Properties of Acetylated Starches from Indian Black Gram (*Phaseolus mungo* L.) Cultivars. J. Food Sci. Technol..

[44] Du S.K., Jiang H.X., Ai Y.F., Jane J.L. (2014). Physicochemical Properties and Digestibility of Common Bean (*Phaseolus vulgaris* L.) Starches. Carbohydr. Polym..

[45] Ma M.T., Wang Y.J., Wang M.X., Jane J.L., Du S.K. (2017). Physicochemical Properties and In Vitro Digestibility of Legume Starches. Food Hydrocolloid.

[46] Andrabi S.N., Wani I.A., Gani A., Hamdani A.M., Masoodi F.A. (2016). Comparative Study of Physico-Chemical and Functional Properties of Starch Extracted from Two Kidney Bean (*Phaseolus vulgaris* L.) and Green Gram Cultivars (*Vigna radiata* L.) Grown in India. Starch-Starke.

[47] Li L.Y., Yuan T.Z., Setia R., Raja R.B., Zhang B., Ai Y.F. (2019). Characteristics of Pea, Lentil and Faba Bean Starches Isolated from Air-Classified Flours in Comparison with Commercial Starches. Food Chem..

[48] Xu M.J., Saleh AS M., Liu Y., Jing L.Z., Zhao K., Wu H., Zhang G.Q., Yang S.O., Li W.H. (2018). The Changes in Structural, Physicochemical, and Digestive Properties of Red Adzuki Bean Starch after Repeated and Continuous Annealing Treatments. Starch-Starke.

[49] Zhang Z.S., Tian X.L., Wang P., Jiang H., Li W.H. (2019). Compositional, Morphological, and Physicochemical Properties of Starches from Red Adzuki Bean, Chickpea, Faba Bean, and Baiyue Bean Grown in China. Food Sci. Nutr..

[50] Wang M., Chen X., Dong L., Nan X., Ji W., Wang S., Sun W., Zhou Q. (2021). Modification of Pea Dietary Fiber by Ultrafine Grinding and Hypoglycemic Effect in Diabetes Mellitus Mice. J. Food Sci..

[51] Mayengbam S., Lambert J.E., Parnell J.A., Tunnicliffe J.M., Nicolucci A.C., Han J., Sturzenegger T., Shearer J., Mickiewicz B., Vogel H.J. (2019). Impact of Dietary Fiber Supplementation on Modulating Microbiota-Host-Metabolic Axes in Obesity. J. Nutr. Biochem..

[52] Brummer Y., Kaviani M., Tosh S.M. (2015). Structural and Functional Characteristics of Dietary Fibre in Beans, Lentils, Peas and Chickpeas. Food Res. Int..

[53] Wu G.J., Liu D., Wan Y.J., Huang X.J., Nie S.P. (2019). Comparison of Hypoglycemic Effects of Polysaccharides from Four Legume Species. Food Hydrocolloid.

[54] Zhang S.J., Hu T.T., Chen Y.Y., Wang S.Y., Kang Y.F. (2020). Analysis of the Polysaccharide Fractions Isolated from Pea (*Pisum sativum* L.) at Different Levels of Purification. J. Food Biochem..

[55] Eslinger A.J., Eller L.K., Reimer R.A. (2014). Yellow Pea Fiber Improves Glycemia and Reduces Clostridium Leptum in Diet-Induced Obese Rats. Nutr. Res..

[56] Lu Z.X., He J.F., Zhang Y.C., Bing D.J. (2020). Composition, Physicochemical Properties of Pea Protein and its Application in Functional Foods. Crit. Rev. Food Sci. Nutr..

[57] Luo X., Fei Y., Xu Q., Lei T., Mo X., Wang Z., Zhang L., Mou X., Li H. (2020). Isolation and Identification of Antioxidant Peptides from Tartary Buckwheat Albumin (*Fagopyrum tataricum* Gaertn.) and their Antioxidant Activities. J. Food Sci..

[58] Popp J., Trendelenburg V., Niggemann B., Randow S., Völker E., Vogel L., Reuter A., Spiric J., Schiller D., Beyer K. (2020). Pea (*Pisum sativum*) Allergy in Children: Pis S 1 is an Immunodominant Major Pea Allergen and Presents Ige Binding Sites with Potential Diagnostic Value. Clin. Exp. Allergy.

[59] Taylor S.L., Marsh J.T., Koppelman S.J., Kabourek J.L., Johnson P.E., Baumert J.L. (2021). A Perspective on Pea Allergy and Pea Allergens. Trends Food Sci. Technol..

[60] Han F., Moughan P.J., Li J.T., Pang S.J. (2020). Digestible Indispensable Amino Acid Scores (Diaas) of Six Cooked Chinese Pulses. Nutrients.

[61] Zhao H.F., Shen C., Wu Z.J., Zhang Z., Xu C.M. (2020). Comparison of Wheat, Soybean, Rice, and Pea Protein Properties for Effective Applications in Food Products. J. Food Biochem..

[62] Hall A.E., Moraru C.I. (2021). Structure and Function of Pea, Lentil and Faba Bean Proteins Treated by High Pressure Processing and Heat Treatment. LWT-Food Sci. Technol..

[63] Ciurescu G., Toncea I., Ropota M., Habeanu M. (2018). Seeds Composition and Their Nutrients Quality of Some Pea (*Pisum sativum* L.) and Lentil (*Lens culinaris* Medik.) Cultivars. Rom. Agric. Res..

[64] Nadeem M.A., Cilesiz Y., Yüce İ., Baloch F.S., Karaköy T. (2021). Macro and Micronutrients Diversity in the Seeds of Field Pea Germplasm. Pak. J. Bot..

[65] Liu D.D., Guan X., Huang K., Li S., Liu J., Yu W.W., Duan R.Q. (2019). Protective Effects of Mung Bean (*Vigna radiata L.*) and Pea (*Pisum sativum* L.) against High-Fat-Induced Oxidative Stress. Food Sci. Nutr..

[66] Padhi E.M.T., Liu R., Hernandez M., Tsao R., Ramdath D.D. (2017). Total Polyphenol Content, Carotenoid, Tocopherol and Fatty Acid Composition of Commonly Consumed Canadian Pulses and their Contribution to Antioxidant Activity. J. Funct. Food.

[67] Gan R.-Y., Wang M.-F., Lui W.-Y., Wu K., Dai S.-H., Sui Z.-Q., Corke H. (2017). Diversity in Antioxidant Capacity, Phenolic Contents, and Flavonoid Contents of 42 Edible Beans from China. Cereal Chem..

[68] Zhao T.Y., Su W.J., Qin Y., Wang L.Y., Kang Y.F. (2020). Phenotypic Diversity of Pea (*Pisum sativum* L.) Varieties and the Polyphenols, Flavonoids, and Antioxidant Activity of their Seeds. Cienc. Rural..

[69] Ma Y., Gao J., Wei Z., Shahidi F. (2021). Effect of in vitro Digestion on Phenolics and Antioxidant Activity of Red and Yellow Colored Pea Hulls. Food Chem..

[70] Borges-Martinez E., Gallardo-Velazquez T., Cardador-Martinez A., Moguel-Concha D., Osorio-Revilla G., Ruiz-Ruiz J.C., Martinez C.J. (2022). Phenolic Compounds Profile and Antioxidant Activity of Pea (*Pisum sativum* L.) and Black Bean (*Phaseolus* L.) sprouts. Food Sci. Technol..

[71] Castaldo L., Izzo L., Gaspari A., Lombardi S., Rodriguez-Carrasco Y., Narvaez A., Grosso M., Ritieni A. (2022). Chemical Composition of Green Pea (*Pisum sativum* L.) Pods Extracts and their Potential Exploitation as Ingredients in Nutraceutical Formulations. Antioxidants.

[72] Abdelghffar E.A., Obaid W.A., Elgamal A.M., Daoud R., Sobeh M., El Raey M.A. (2021). Pea (*Pisum sativum*) Peel Extract Attenuates Dox-Induced Oxidative Myocardial Injury. Biomed. Pharm..

[73] Guo F.H., Tsao R., Wang X.Y., Jiang L., Sun Y., Xiong H. (2021). Phenolics of Yellow Pea (*Pisum sativum* L.) Hulls, their Plasma and Urinary Metabolites, Organ Distribution, and In Vivo Antioxidant Activities. J. Agric. Food Chem..

[74] Chahbani A., Fakhfakh N., Balti M.A., Mabrouk M., El-Hatmi H., Zouari N., Kechaou N. (2018). Microwave Drying Effects on Drying Kinetics, Bioactive Compounds and Antioxidant Activity of Green Peas (*Pisum sativum* L.). Food Biosci..

[75] Elessawy F.M., Bazghaleh N., Vandenberg A., Purves R.W. (2020). Polyphenol Profile Comparisons of Seed Coats of Five Pulse Crops Using a Semi-Quantitative Liquid Chromatography-Mass Spectrometric Method. Phytochem. Anal..

[76] Jha A.B., Purves R.W., Elessawy F.M., Zhang H.X., Vandenberg A., Warkentin T.D. (2019). Polyphenolic Profile of Seed Components of White and Purple Flower Pea Lines. Crop. Sci..

[77] Guo F.H., Tsao R., Li C.Y., Wang X.Y., Zhang H., Jiang L., Sun Y., Xiong H. (2021). Green Pea (*Pisum sativum* L.) Hull Polyphenol Extracts Ameliorate Dss-Induced Colitis through Keap1/Nrf2 Pathway and Gut Microbiota Modulation. Foods.

[78] Mejri F., Ben Khoud H., Njim L., Baati T., Selmi S., Martins A., Serralheiro M.L.M., Rauter A.P., Hosni K. (2019). In Vitro and In Vivo Biological Properties of Pea Pods (*Pisum sativum* L.). Food Biosci..

[79] Carpentier J., Conforto E., Chaigneau C., Vendeville J.E., Maugard T. (2022). Microencapsulation and Controlled Release of Alpha-Tocopherol by Complex Coacervation between Pea Protein and Tragacanth Gum: A Comparative Study with Arabic and Tara Gums. Innov. Food Sci. Emerg. Technol..

[80] Nazir N., Nisar M., Ahmad S., Wadood S.F., Jan T., Zahoor M., Ahmad M., Ullah A. (2020). Characterization of Phenolic Compounds in Two Novel Lines of *Pisum sativum* L. Along with their in vitro Antioxidant Potential. Environ. Sci. Pollut. Res..

[81] Troszynska A., Ciska E. (2002). Phenolic Compounds of Seed Coats of White and Coloured Varieties of Pea (*Pisum sativum* L.) and their Total Antioxidant Activity. Czech J. Food Sci..

[82] Costantini M., Summo C., Centrone M., Rybicka I., D’agostino M., Annicchiarico P., Caponio F., Pavan S., Tamma G., Pasqualone A. (2021). Macro- and Micro-Nutrient Composition and Antioxidant Activity of Chickpea and Pea Accessions. Pol. J. Food Nutr. Sci..

[83] Sharma A. (2021). A Review on Traditional Technology and Safety Challenges with Regard to Antinutrients in Legume Foods. J. Food Sci. Technol..

[84] Sinkovic L., Pipan B., Sibul F., Nemes I., Horecki A.T., Meglic V. (2023). Nutrients, Phytic Acid and Bioactive Compounds in Marketable Pulses. Plants.

[85] Hugman J., Wang L.F., Beltranena E., Htoo J.K., Zijlstra R.T. (2021). Nutrient Digestibilityof Heat-Processed Field Pea in Weaned Pigs. Anim. Feed. Sci. Technol..

[86] Moore K.L., Rodríguez-Ramiro I., Jones E.R., Jones E.J., Rodríguez-Celma J., Halsey K., Domoney C., Shewry P.R., Fairweather-Tait S., Balk J. (2018). The Stage of Seed Development Influences Iron Bioavailability in Pea (*Pisum sativum* L.). Sci. Rep..

[87] Xu B., Chang S.K.C. (2012). Comparative Study on Antiproliferation Properties and Cellular Antioxidant Activities of Commonly Consumed Food Legumes against Nine Human Cancer Cell Lines. Food Chem..

[88] Kamalasundari S., Babu R., Umamaheswari T. (2019). Effect of Domestic Processing Methods on Anti-Nutritional Factors and its Impact on the Bio-Availability Proteins and Starch in Commonly Consumed Whole Legumes. Asian J. Dairy. Food Res..

[89] Moussou N., Ouazib M., Wanasundara J., Zaidi F., Rubio L.A. (2019). Nutrients and Non-Nutrients Composition and in vitro Starch Digestibility of Five Algerian Legume Seed Flours. Int. Food Res. J..

[90] Sharma K., Kumar V., Kaur J., Tanwar B., Goyal A., Sharma R., Gat Y., Kumar A. (2021). Health Effects, Sources, Utilization and Safety of Tannins: A Critical Review. Toxin Rev..

[91] Fraga-Corral M., Otero P., Echave J., Garcia-Oliveira P., Carpena M., Jarboui A., Nuñez-Estevez B., Simal-Gandara J., Prieto M.A. (2021). By-Products of Agri-Food Industry as Tannin-Rich Sources: A Review Of Tannins’ Biological Activities and their Potential for Valorization. Foods.

[92] Ge G., Guo W.X., Zheng J.B., Zhao M.M., Sun W.Z. (2021). Effect of Interaction between Tea Polyphenols with Soymilk Protein on Inactivation of Soybean Trypsin Inhibitor. Food Hydrocolloid.

[93] Zhou J.J., Li M.H., Bai Q., de Souza T.S.P., Barrow C., Dunshea F., Suleria H.A.R. (2023). Effects of Different Processing Methods on Pulses Phytochemicals: An Overview. Food Rev. Int..

[94] Espinosa D.C.P., Cortina J.R., Carrión M.H., Mora O.O. (2021). Drying and Cooking Effects on the Final Quality of Pea Grains (*Pisum sativum* L.) Varieties. Food Sci. Technol..

[95] Yu F., Yang Z., Tao Z.C., Yang Z.Y. (2020). Optimization of Pea Seed Intermittent Drying Assisted with Ultrasound Technology. Int. J. Food Eng..

[96] Yang Z., Li X., Tao Z.C., Luo N., Yu F. (2018). Ultrasound-Assisted Heat Pump Drying of Pea Seed. Dry. Technol..

[97] Cappelli A., Oliva N., Cini E. (2020). Stone Milling Versus Roller Milling: A Systematic Review of the Effects on Wheat Flour Quality, Dough Rheology, and Bread Characteristics. Trends Food Sci. Technol..

[98] Gu Z.X., Jiang H.Y., Zha F.C., Manthey F., Rao J.J., Chen B.C. (2021). Toward a Comprehensive Understanding of Ultracentrifugal Milling on the Physicochemical Properties and Aromatic Profile of Yellow Pea Flour. Food Chem..

[99] Schmidt F., Blankart M., Wanger J., Scharfe M., Scheuerer T., Hinrichs J. (2022). Upscaling of Alkaline Pea Protein Extraction from Dry Milled and Pre-Treated Peas from Laboratory to Pilot Scale: Optimization of Process Parameters for Higher Protein Yields. J. Food Meas. Charact..

[100] Kaiser A.C., Barber N., Manthey F., Hall C. (2019). Physicochemical Properties of Hammer-Milled Yellow Split Pea (*Pisum sativum* L.). Cereal Chem..

[101] Nguyen G.T., Gidley M.J., Sopade P.A. (2015). Dependence of In-Vitro Starch and Protein Digestions on Particle Size of Field Peas (*Pisum sativum* L.). LWT-Food Sci. Technol..

[102] Li H., Zou L., Li X.Y., Wu D.T., Liu H.Y., Li H.B., Gan R.Y. (2022). Adzuki Bean (*Vigna angularis*): Chemical Compositions, Physicochemical Properties, Health Benefits, and Food Applications. Compr. Rev. Food Sci. Food Saf..

[103] Setia R., Dai Z.X., Nickerson M.T., Sopiwnyk E., Malcolmson L., Ai Y.F. (2019). Impacts of Short-Term Germination on the Chemical Compositions, Technological Characteristics and Nutritional Quality of Yellow Pea and Faba Bean Flours. Food Res. Int..

[104] Shi L., Mu K.W., Arntfield S.D., Nickerson M.T. (2017). Changes in Levels of Enzyme Inhibitors during Soaking and Cooking for Pulses Available in Canada. J. Food Sci. Technol..

[105] Wang N., Hatcher D.W., Gawalko E.J. (2008). Effect of Variety and Processing on Nutrients and Certain Anti-Nutrients in Field Peas (*Pisum sativum*). Food Chem..

[106] Skalickova S., Ridoskova A., Slama P., Skladanka J., Skarpa P., Smykalova I., Horacek J., Dostalova R., Horky P. (2022). Effect of Lactic Fermentation and Cooking on Nutrient and Mineral Digestibility of Peas. Front. Nutr..

[107] Stone A.K., Waelchli K.N., Cabuk B., Mcintosh T.C., Wanasundara J., Arntfield S.D., Nickerson M.T. (2021). The Levels of Bioactive Compounds Found in Raw and Cooked Canadian Pulses. Food Sci. Technol. Int..

[108] Liu Y.H., Ragaee S., Marcone M.F., Abdel-Aal E.M. (2020). Composition of Phenolic Acids and Antioxidant Properties of Selected Pulses Cooked with Different Heating Conditions. Foods.

[109] Shin J.A., Heo Y., Seo M., Choi Y., Lee K.T. (2016). Effects of Cooking Methods on the Beta-Carotene Levels of Selected Plant Food Materials. Food Sci. Biotechnol..

[110] Liu Y.H., Ragaee S., Marcone M.F., Abdel-Aal E.M. (2020). Effect of Different Cooking Methods and Heating Solutions on Nutritionally-Important Starch Fractions and Flatus Oligosaccharides in Selected Pulses. Cereal Chem..

[111] Obadi M., Xu B. (2021). Review on the Physicochemical Properties, Modifications, and Applications of Starches and its Common Modified Forms Used in Noodle Products. Food Hydrocolloid.

[112] He X., Dai T., Liang R., Liu W., Cheng Y., Liu C., Chen J. (2023). A New Partially-Gelatinized Granular Starch Prepared by Industry-Scale Microfluidization Treatment of Pea Starch. Innov. Food Sci. Emerg. Technol..

[113] Shi M.M., Gao Q.Y., Liu Y.Q. (2018). Changes in the Structure and Digestibility of Wrinkled Pea Starch with Malic Acid Treatment. Polymers.

[114] Han L.H., Cao S.P., Yu Y.T., Xu X.C., Cao X.H., Chen W.J. (2021). Modification in Physicochemical, Structural and Digestive Properties of Pea Starch during Heat-Moisture Process Assisted by Pre- and Post-Treatment of Ultrasound. Food Chem..

[115] Majeed T., Majeed T., Wani I.A., Wani I.A., Hamdani A.M., Hamdani A.M., Bhat N.A., Bhat N.A., Majeed T., Majeed T. (2018). Effect of Sonication and Gamma-Irradiation on the Properties of Pea (*Pisum sativum*) and Vetch (*Vida villosa*) Starches: A Comparative Study. Int. J. Biol. Macromol..

[116] Zhou D.T., Ma Z., Yin X.X., Hu X.Z., Boye J.I. (2019). Structural Characteristics and Physicochemical Properties of Field Pea Starch Modified by Physical, Enzymatic, and Acid Treatments. Food Hydrocolloid.

[117] Chen X.Y., Zhang Z.W., Ji N., Li M., Wang Y.F., Xiong L., Sun Q.J. (2022). The Effect of Ethanol Solution Annealing on the Physicochemical Properties of Pea and Potato Starches. Food Hydrocolloid.

[118] Yan Y.Z., Peng B.X., Niu B., Ji X.L., He Y., Shi M.M. (2022). Understanding the Structure, Thermal, Pasting, and Rheological Properties of Potato and Pea Starches Affected by Annealing Using Plasma-Activated Water. Front. Nutr..

[119] Arteaga V.G., Guardia M.A., Muranyi I., Eisner P., Schweiggert-Weisz U. (2020). Effect of Enzymatic Hydrolysis on Molecular Weight Distribution, Techno- Functional Properties and Sensory Perception of Pea Protein Isolates. Innov. Food Sci. Emerg. Technol..

[120] Vatansever S., Ohm J.B., Simsek S., Hall C. (2022). A Novel Approach: Supercritical Carbon Dioxide Plus Ethanol Extraction to Improve Techno-Functionalities of Pea Protein Isolate. Cereal Chem..

[121] Wang F., Zhang Y.Z., Xu L., Ma H. (2020). An Efficient Ultrasound-Assisted Extraction Method of Pea Protein and its Effect on Protein Functional Properties and Biological Activities. LWT-Food Sci. Technol..

[122] Ozturk O.K., Turasan H. (2022). Latest Developments in the Applications of Microfluidization to Modify the Structure of Macromolecules Leading to Improved Physicochemical and Functional Properties. Crit. Rev. Food Sci. Nutr..

[123] He X.H., Chen J., He X.M., Feng Z., Li C.H., Liu W., Dai T.T., Liu C.M. (2021). Industry-Scale Microfluidization as a Potential Technique to Improve Solubility and Modify Structure of Pea Protein. Innov. Food Sci. Emerg. Technol..

[124] Oliete B., Potin F., Cases E., Saurel R. (2019). Microfluidization as Homogenization Technique in Pea Globulin-Based Emulsions. Food Bioprocess. Technol..

[125] Stanisavljevic N.S., Ilic M., Jovanovic Z.S., Cupic T., Dabic D.C., Natic M.M., Tesic Z.L., Radovic S.S. (2015). Identification of Seed Coat Phenolic Compounds from Differently Colored Pea Varieties and Characterization of their Antioxidant Activity. Arch. Biol. Sci..

[126] Stanisavljevic N.S., Ilic M.D., Matic I.Z., Jovanovic Z.S., Cupic T., Dabic D.C., Natic M.M., Tesic Z.L. (2016). Identification of Phenolic Compounds from Seed Coats of Differently Colored European Varieties of Pea (*Pisum sativum* L.) and Characterization of their Antioxidant and In Vitro Anticancer Activities. Nutr. Cancer.

[127] Hadrich F., El Arbi M., Boukhris M., Sayadi S., Cherif S. (2014). Valorization of the Peel of Pea: *Pisum sativum* by Evaluation of its Antioxidant and Antimicrobial Activities. J. Oleo Sci..

[128] Jalili Safaryan M., Ganjloo A., Bimakr M., Zarringhalami S. (2016). Optimization of Ultrasound-Assisted Extraction, Preliminary Characterization and In Vitro Antioxidant Activity of Polysaccharides from Green Pea Pods. Foods.

[129] Ding J., Liang R., Yang Y.Y., Sun N., Lin S.Y. (2020). Optimization of Pea Protein Hydrolysate Preparation and Purification of Antioxidant Peptides Based on an In Silico Analytical Approach. LWT-Food Sci. Technol..

[130] Guo F.H., Xiong H., Wang X.Y., Jiang L., Yu N.X., Hu Z.Y., Sun Y., Tsao R. (2019). Phenolics of Green Pea (*Pisum sativum* L.) Hulls, their Plasma and Urinary Metabolites, Bioavailability, and In Vivo Antioxidant Activities in a Rat Model. J. Agric. Food Chem..

[131] Guo F.H., Tsao R., Li C.Y., Wang X.Y., Zhang H., Jiang L., Sun Y., Xiong H. (2022). Polyphenol Content of Green Pea (*Pisum sativum* L.) Hull Under In Vitro Digestion and Effects of Digestive Products on Anti-Inflammatory Activity and Intestinal Barrier in the Caco-2/Raw264.7 Coculture Model. J. Agric. Food Chem..

[132] Ndiaye F., Vuong T., Duarte J., Aluko R.E., Matar C. (2012). Anti-Oxidant, Anti-Inflammatory and Immunomodulating Properties of an Enzymatic Protein Hydrolysate from Yellow Field Pea Seeds. Eur. J. Nutr..

[133] Bibi S., Moraes LF D., Lebow N., Zhu M.J. (2017). Dietary Green Pea Protects against Dss-Induced Colitis in Mice Challenged with High-Fat Diet. Nutrients.

[134] Utrilla M.P., Peinado M.J., Ruiz R., Rodriguez-Nogales A., Algieri F., Rodriguez-Cabezas M.E., Clemente A., Galvez J., Rubio L.A. (2015). Pea (*Pisum sativum* L.) Seed Albumin Extracts Show Anti-Inflammatory Effect in the Dss Model of Mouse Colitis. Mol. Nutr. Food Res..

[135] Jakubczyk A., Baraniak B. (2014). Angiotensin I Converting Enzyme Inhibitory Peptides Obtained after in vitro Hydrolysis of Pea (*Pisum sativum* Var. Bajka) Globulins. Biomed. Res. Int..

[136] Liao W., Fan H.B., Liu P., Wu J.P. (2019). Identification of Angiotensin Converting Enzyme 2 (Ace2) Up-Regulating Peptides from Pea Protein Hydrolysate. J. Funct. Food.

[137] Wang X., Bhullar K.S., Fan H.B., Liao W., Qiao Y.J., Su D., Wu J.P. (2020). Regulatory Effects of a Pea-Derived Peptide Leu-Arg-Trp (Lrw) on Dysfunction of Rat Aortic Vascular Smooth Muscle Cells Against Angiotensin Ii Stimulation. J. Agric. Food Chem..

[138] Girgih A.T., Nwachukwu I.D., Onuh J.O., Malomo S.A., Aluko R.E. (2016). Antihypertensive Properties of a Pea Protein Hydrolysate during Short-and Long-Term Oral Administration to Spontaneously Hypertensive Rats. J. Food Sci..

[139] Inagaki K., Nishimura Y., Iwata E., Manabe S., Goto M., Ogura Y., Hotta H. (2016). Hypolipidemic Effect of the Autoclaved Extract Prepared from Pea (*Pisum sativum* L.) Pods In Vivo and In Vitro. J. Nutr. Sci. Vitam..

[140] Rigamonti E., Parolini C., Marchesi M., Diani E., Brambilla S., Sirtori C.R., Chiesa G. (2010). Hypolipidemic Effect of Dietary Pea Proteins: Impact on Genes Regulating Hepatic Lipid Metabolism. Mol. Nutr. Food Res..

[141] Ruiz R., Olias R., Clemente A., Rubio L.A. (2020). A Pea (*Pisum sativum* L.) Seed Vicilins Hydrolysate Exhibits Ppar Gamma Ligand Activity and Modulates Adipocyte Differentiation in a 3t3-L1 Cell Culture Model. Foods.

[142] Awosika T.O., Aluko R.E. (2019). Inhibition of the in vitro Activities of Alpha-Amylase, Alpha-Glucosidase and Pancreatic Lipase by Yellow Field Pea (*Pisum sativum* L.) Protein Hydrolysates. Int. J. Food Sci. Technol..

[143] Qin G., Xu W., Liu J., Zhao L., Chen G. (2021). Purification, Characterization and Hypoglycemic Activity of Glycoproteins Obtained from Pea (*Pisum sativum* L.). Food Sci. Hum. Wellness.

[144] Liu J.P., Qian Y.F., Qin GY X., Zhao L.Y., Chen G.T. (2021). Antidiabetic Activities of Glycoprotein from Pea (*Pisum sativum* L.) in Stz-Induced Diabetic Mice. Food Funct..

[145] Wei Y., Zhang R.X., Fang L., Qin X.Y., Cai M.Y., Gu R.Z., Lu J., Wang Y.Q. (2019). Hypoglycemic Effects and Biochemical Mechanisms of Pea Oligopeptide on High-Fat Diet and Streptozotocin-Induced Diabetic Mice. J. Food Biochem..

[146] Thondre P.S., Achebe I., Sampson A., Maher T., Guerin-Deremaux L., Lefranc-Millot C., Ahlstrom E., Lightowler H. (2021). Co-Ingestion of Nutralys^®^ Pea Protein and a High-Carbohydrate Beverage Influences the Glycaemic, Insulinaemic, Glucose-Dependent Insulinotropic Polypeptide (Gip) and Glucagon-Like Peptide-1 (Glp-1) Responses: Preliminary Results of a Randomised Controlled Trial. Eur. J. Nutr..

[147] El-Saadony M.T., El-Hack M.E.A., Swelum A.A., Al-Sultan S.I., El-Ghareeb W.R., Hussein E.O.S., Ba-Awadh H.A., Akl B.A., Nader M.M. (2021). Enhancing Quality and Safety of Raw Buffalo Meat Using the Bioactive Peptides of Pea and Red Kidney Bean under Refrigeration Conditions. Ital. J. Anim. Sci..

[148] El-Araby M.M., El-Shatoury E.H., Soliman M.M., Shaaban H.F. (2020). Characterization and Antimicrobial Activity of Lectins Purified from Three Egyptian Leguminous Seeds. Amb. Express.

[149] Belghith-Fendri L., Chaari F., Ben Jeddou K., Kallel F., Bouaziz F., Helbert C.B., Abdelkefi-Mesrati L., Ellouz-Chaabouni S., Ghribi-Aydi D. (2018). Identification of Polysaccharides Extracted from Pea Pod By-Products and Evaluation of their Biological and Functional Properties. Int. J. Biol. Macromol..

[150] Hidayat M., Prahastuti S., Afifah E., Widowati W., Yusuf M., Hasan K. (2022). The Role of Green Peas Protein Hydrolysate in Tgf/Smad Signaling to Prevent Renal Fibrosis. J. King Saud. Univ. Sci..

[151] Hidayat M., Prahastuti S., Yusuf M., Hasan K. (2021). Nutrition Profile and Potency of Rgd Motif in Protein Hydrolysate of Green Peas as an Antifibrosis in Chronic Kidney Disease. Iran. J. Basic. Med. Sci..

[152] Kabir S.R., Nabi M.M., Haque A., Zaman R.U., Mahmud Z.H., Abu Reza M. (2013). Pea Lectin Inhibits Growth of Ehrlich Ascites Carcinoma Cells by Inducing Apoptosis and G_2_/M Cell Cycle Arrest In Vivo in Mice. Phytomedicine.

[153] Arora H., Shang N., Bhullar K.S., Wu J.P. (2020). Pea Protein-Derived Tripeptide Lrw Shows Osteoblastic Activity on Mc3t3-E1 Cells via the Activation of the Akt/Runx2 Pathway. Food Funct..

[154] Feng T., Huang Y.Y., Tang Z.H., Wei D.D., Mo J.M. (2021). Anti-Fatigue Effects of Pea (*Pisum sativum* L.) Peptides Prepared by Compound Protease. J. Food Sci. Technol..

[155] Liu Y.P., Su Y.Y., Yang Q., Zhou T.B. (2021). Stem Cells in the Treatment Of Renal Fibrosis: A Review of Preclinical and Clinical Studies of Renal Fibrosis Pathogenesis. Stem Cell Res. Ther..

[156] Bi S., Xu X., Luo D., Lao F., Pang X., Shen Q., Hu X., Wu J. (2020). Characterization of Key Aroma Compounds in Raw and Roasted Peas (*Pisum sativum* L.) by Application of Instrumental and Sensory Techniques. J. Agric. Food Chem..

[157] Trikusuma M., Paravisini L., Peterson D.G. (2020). Identification of Aroma Compounds in Pea Protein Uht Beverages. Food Chem..

[158] Bi S., Lao F., Pan X., Shen Q., Liu Y., Wu J.H. (2022). Flavor Formation and Regulation of Peas (*Pisum sativum* L.) Seed Milk via Enzyme Activity Inhibition and Off-Flavor Compounds Control Release. Food Chem..

[159] Ma W.Y., Zhang C.M., Kong X.Z., Li X.F., Chen Y.M., Hua Y.F. (2021). Effect of Pea Milk Preparation on the Quality of Non-Dairy Yoghurts. Food Biosci..

[160] Manus J., Millette M., Dridi C., Salmieri S., Uscanga BR A., Lacroix M. (2021). Protein Quality of a Probiotic Beverage Enriched with Pea and Rice Protein. J. Food Sci..

[161] Yousseef M., Lafarge C., Valentin D., Lubbers S., Husson F. (2016). Fermentation of Cow Milk and/or Pea Milk Mixtures by Different Starter Cultures: Physico-Chemical and Sensorial Properties. LWT-Food Sci. Technol..

[162] Sim S.Y.J., Hua X.Y., Henry C.J. (2020). A Novel Approach to Structure Plant-Based Yogurts Using High Pressure Processing. Foods.

[163] Nawaz M.A., Tan M.V., Oiseth S., Buckow R. (2022). An Emerging Segment of Functional Legume-Based Beverages: A Review. Food Rev. Int..

[164] Gan R.Y., Lui W.Y., Wu K., Chan C.L., Dai S.H., Sui Z.Q., Corke H. (2017). Bioactive Compounds and Bioactivities of Germinated Edible Seeds and Sprouts: An Updated Review. Trends Food Sci. Technol..

[165] Erba D., Angelino D., Marti A., Manini F., Faoro F., Morreale F., Pellegrini N., Casiraghi M.C. (2019). Effect of Sprouting on Nutritional Quality of Pulses. Int. J. Food Sci. Nutr..

[166] Elliott H., Woods P., Green B.D., Nugent A.P. (2022). Can Sprouting Reduce Phytate and Improve the Nutritional Composition and Nutrient Bioaccessibility in Cereals and Legumes?. Nutr. Bull..

[167] Ekezie F.-G.C., Sun D.-W., Cheng J.-H. (2017). A Review on Recent Advances in Cold Plasma Technology for the Food Industry: Current Applications and Future Trends. Trends Food Sci. Technol..

[168] Svubova R., Kyzek S., Medvecka V., Slovakova L., Galova E., Zahoranova A. (2020). Novel Insight at the Effect of Cold Atmospheric Pressure Plasma on the Activity of Enzymes Essential for the Germination of Pea (*Pisum sativum* L. Cv. Prophet) Seeds. Plasma Chem. Plasma Process..

[169] Zhang C.L., Zhang Y.Y., Zhao Z.Y., Liu W.F., Chen Y.Q., Yang G.J., Xia X.D., Cao Y.F. (2019). The Application of Slightly Acidic Electrolyzed Water in Pea Sprout Production to Ensure Food Safety, Biological and Nutritional Quality of the Sprout. Food Control..

[170] Jerse A., Kacjan-Marsic N., Sircelj H., Germ M., Kroflic A., Stibilj V. (2017). Seed Soaking in I and Se Solutions Increases Concentrations of Both Elements and Changes Morphological and Some Physiological Parameters of Pea Sprouts. Plant. Physiol. Biochem..

[171] Baczek-Kwinta R., Baran A., Simlat M., Lang J., Bieniek M., Florek B. (2020). Enrichment of Different Plant Seeds with Zinc and Assessment of Health Risk of Zn-Fortified Sprouts Consumption. Agronomy.

[172] Tan C., Zhang L., Duan X., Chai X., Huang R., Kang Y., Yang X. (2022). Effects of Exogenous Sucrose and Selenium on Plant Growth, Quality, and Sugar Metabolism of Pea Sprouts. J. Sci. Food Agric..

[173] Zhang Y.Q., Hu R.J., Tilley M., Siliveru K., Li Y.H. (2021). Effect of Pulse Type and Substitution Level on Dough Rheology and Bread Quality of Whole Wheat-Based Composite Flours. Processes.

[174] Kotsiou K., Sacharidis D.D., Matsakidou A., Biliaderis C.G., Lazaridou A. (2021). Impact of Roasted Yellow Split Pea Flour on Dough Rheology and Quality of Fortified Wheat Breads. Foods.

[175] Millar K.A., Barry-Ryan C., Burke R., Mccarthy S., Gallagher E. (2019). Dough Properties and Baking Characteristics of White Bread, as Affected by Addition of Raw, Germinated and Toasted Pea Flour. Innov. Food Sci. Emerg. Technol..

[176] Millar K.A., Barry-Ryan C., Burke R., Hussey K., Mccarthy S., Gallagher E. (2017). Effect of Pulse Flours on the Physiochemical Characteristics and Sensory Acceptance of Baked Crackers. Int. J. Food Sci. Technol..

[177] Krause S., Debon S., Palchen K., Jakobi R., Rega B., Bonazzi C., Grauwet T. (2022). In Vitro Digestion of Protein and Starch in Sponge Cakes Formulated with Pea (*Pisum sativum* L.) Ingredients. Food Funct..

[178] Hanan E., Rudra S.G., Sagar V.R., Sharma V. (2020). Utilizationof Pea Pod Powder for Formulation of Instant Pea Soup Powder. J. Food Process. Preserv..

[179] Polizer-Rocha Y.J., Lorenzo J.M., Pompeu D., Rodrigues I., Baldin J.C., Pires M.A., Freire MT A., Barba F.J., Trindade M.A. (2020). Physicochemical and Technological Properties of Beef Burger as Influenced by the Addition of Pea Fibre. Int. J. Food Sci. Technol..

[180] Pietrasik Z., Sigvaldson M., Soladoye O.P., Gaudette N.J. (2020). Utilization of Pea Starch and Fibre Fractions for Replacement of Wheat Crumb in Beef Burgers. Meat Sci..

[181] Baugreet S., Kerry J.P., Botinestean C., Allen P., Hamill R.M. (2016). Development of Novel Fortified Beef Patties with Added Functional Protein Ingredients for the Elderly. Meat Sci..

[182] Shoaib A., Sahar A., Sameen A., Saleem A., Tahir A.T. (2018). Use of Pea and Rice Protein Isolates as Source of Meat Extenders in the Development of Chicken Nuggets. J. Food Process. Preserv..

[183] Osen R., Toelstede S., Eisner P., Schweiggert-Weisz U. (2015). Effect of High Moisture Extrusion Cooking on Protein-Protein Interactions of Pea (*Pisum sativum* L.) Protein Isolates. Int. J. Food Sci. Technol..

[184] Golge O., Kilincceker O., Koluman A. (2018). Effects of Different Fibers on The Quality of Chicken Meatballs. J. Food Saf. Food Qual..

[185] Kehlet U., Pagter M., Aaslyng M.D., Raben A. (2017). Meatballs with 3% and 6% Dietary Fibre from Rye Bran or Pea Fibre—Effects on Sensory Quality and Subjective Appetite Sensations. Meat Sci..

[186] Hadidi M., Boostani S., Jafari S.M. (2022). Pea Proteins as Emerging Biopolymers for the Emulsification and Encapsulation of Food Bioactives. Food Hydrocolloid.

[187] Caballero S., Li Y.O., Mcclements D.J., Davidov-Pardo G. (2022). Hesperetin (Citrus Peel Flavonoid Aglycone) Encapsulation Using Pea Protein-High Methoxyl Pectin Electrostatic Complexes: Complex Optimization and Biological Activity. J. Sci. Food Agric..

[188] Li J.T., Zhang X.G., Zhao R., Lu Y.C., Wang C., Wang C.N. (2022). Encapsulation of Quercetin in Pea Protein-High Methoxyl Pectin Nanocomplexes: Formation, Stability, Antioxidant Capacity and In Vitro Release Profile. Food Biosci..

[189] Okagu O.D., Udenigwe C.C. (2022). Molecular Interactions of Pea Globulin, Albumin and Glutelin with Curcumin: Formation and Gastric Release Mechanisms of Curcumin-Loaded Bio-Nanocomplexes. Food Biophys..

[190] Zhang X.G., Wang C., Qi Z.T., Zhao R., Wang C.N., Zhang T.H. (2022). Pea Protein Based Nanocarriers for Lipophilic Polyphenols: Spectroscopic Analysis, Characterization, Chemical Stability, Antioxidant and Molecular Docking. Food Res. Int..

[191] Mihalca V., Kerezsi A.D., Weber A., Gruber-Traub C., Schmucker J., Vodnar D.C., Dulf F.V., Socaci S.A., Fărcaș A., Mureșan C.I. (2021). Protein-Based Films and Coatings for Food Industry Applications. Polymers.

[192] Cheng J.J., Li Z.Z., Wang J., Zhu Z.B., Yi J.H., Chen B.C., Cui L.Q. (2022). Structural Characteristics of Pea Protein Isolate (PPI) Modified by High-Pressure Homogenization and its Relation to the Packaging Properties of Ppi Edible Film. Food Chem..

[193] Azevedo V.M., De Oliveira A.C.S., Borges S.V., Raguzzoni J.C., Dias M.V., Costa A.L.R. (2020). Pea Protein Isolate Nanocomposite Films for Packaging Applications: Effect of Starch Nanocrystals on the Structural, Morphological, Thermal, Mechanical and Barrier Properties. Emir. J. Food Agric..

[194] Li H., Shi H.B., He Y.Q., Fei X., Peng L.C. (2020). Preparation and Characterization of Carboxymethyl Cellulose-Based Composite Films Reinforced by Cellulose Nanocrystals Derived from Pea Hull Waste for Food Packaging Applications. Int. J. Biol. Macromol..

[195] Salim M.H., Abdellaoui Y., Benhamou A.A., Ablouh E.H., El Achaby M., Kassab Z. (2022). Influence of Cellulose Nanocrystals from Pea Pod Waste on Mechanical, Thermal, Biodegradability, and Barrier Properties of Chitosan-Based Films. Cellulose.

[196] Zhou X., Cheng R., Wang B., Zeng J., Xu J., Li J., Kang L., Cheng Z., Gao W., Chen K. (2021). Biodegradable Sandwich-Architectured Films Derived from Pea Starch and Polylactic Acid with Enhanced Shelf-Life for Fruit Preservation. Carbohydr. Polym..

[197] Zhou X.M., Yang R.D., Wang B., Chen K.F. (2019). Development and Characterization of Bilayer Films Based on Pea Starch/Polylactic Acid and Use in the Cherry Tomatoes Packaging. Carbohydr. Polym..

